# Bidirectional association between perioperative skeletal muscle and subcutaneous fat in colorectal cancer patients and their prognostic significance

**DOI:** 10.3389/fnut.2024.1381995

**Published:** 2024-09-18

**Authors:** Guanghong Yan, Lizhu Liu, Mengmei Liu, Xinyue Jiang, Ping Chen, Min Li, Qingyan Ma, Yani Li, Sifan Duan, Ruimin You, Yanni Huang, Zhenhui Li, Dingyun You

**Affiliations:** ^1^Yunnan Provincial Key Laboratory of Public Health and Biosafety, School of Public Health, Kunming Medical University, Kunming, China; ^2^Department of Radiology, The Third Affiliated Hospital of Kunming Medical University, Yunnan Cancer Hospital, Yunnan Cancer Center, Kunming, China; ^3^Second Ward of Gastrointestinal Surgery, The Second Affiliated Hospital of Kunming Medical University, Kunming, China

**Keywords:** skeletal muscle index, subcutaneous fat index, RI-CLPM, colorectal cancer, retrospective cohort

## Abstract

**Introduction:**

Low skeletal muscle mass and high adipose tissue coexist across the body weight spectrum and independently predict the survival ratio of colorectal cancer (CRC) patients. This combination may lead to a mutually exacerbating vicious cycle. Tumor-associated metabolic conditions primarily affect subcutaneous adipose tissue, but the nature and direction of its relationship with skeletal muscle are unclear. This study aims to examine the bidirectional causal relationship between skeletal muscle index (SMI) and subcutaneous fat index (SFI) during the perioperative period in CRC patients; as well as to validate the association between perioperative SMI, SFI, and CRC prognosis.

**Methods:**

This population-based retrospective cohort study included patients with stage I-III colorectal cancer who underwent radical resection at the Third Affiliated Hospital of Kunming Medical University between September 2012 and February 2019. Based on inclusion and exclusion criteria, 1,448 patients were analyzed. Preoperative (P1), 2 months postoperative (P2), and 5 months postoperative (P3) CT scans were collected to evaluate the skeletal muscle index (SMI; muscle area at the third lumbar vertebra divided by height squared) and subcutaneous fat index (SFI; subcutaneous fat area at the third lumbar vertebra divided by height squared). A random intercept cross-lagged panel model (RI-CLPM) was used to examine the intra-individual relationship between SMI and SFI, and Cox regression was employed to assess the association between SMI, SFI, recurrence-free survival (RFS), and overall survival (OS).

**Results:**

The median age at diagnosis was 59.00 years (IQR: 51.00–66.00), and 587 patients (40.54%) were female. RI-CLPM analysis revealed a negative correlation between SFI and subsequent SMI at the individual level: P1-P2 (*β* = −0.372, *p* = 0.038) and P2-P3 (*β* = −0.363, *p* = 0.001). SMI and SFI showed a negative correlation during P1-P2 (*β* = −0.363, *p* = 0.001) but a positive correlation during P2-P3 (*β* = 0.357, *p* = 0.006). No significant correlation was found between the random intercepts of SFI and SMI at the between-person level (*r* = 0.157, *p* = 0.603). The Cox proportional hazards multivariate regression model identified that patients with elevated SFI had poorer recurrence-free survival (HR, 1.24; 95% CI: 1.00–1.55). Compared to patients with normal preoperative SMI and SFI, those with low SMI or high SFI had poorer recurrence-free survival (HR, 1.26; 95% CI: 1.03–1.55) and overall survival (HR, 1.39; 95% CI: 1.04–1.87). However, no significant association between SMI and SFI and the prognosis of colorectal cancer patients was observed postoperatively.

**Conclusion:**

In CRC patients, preoperative muscle loss leads to postoperative fat accumulation, exacerbating muscle loss in a feedback loop. Elevated preoperative SFI predicts poorer survival outcomes. Monitoring SMI and SFI is crucial as prognostic indicators, despite non-significant postoperative associations. Further research is needed to improve patient outcomes.

## 1 Introduction

Colorectal cancer (CRC) is the third most common malignant tumor and the second leading cause of cancer deaths worldwide (1). The number of patients with CRC increases with changing lifestyles (2). One-third of CRC patients are malnourished, and this proportion reaches 65% in patients with metastatic colorectal cancer (3, 4). Nutrient intake and consumption are closely related to changes in body composition. Cancer patients, including those with CRC, often undergo abnormal body composition changes due to inadequate food intake, reduced physical activity, and catabolic disorders (5). In addition, abnormal body composition is a risk factor for poor prognosis in CRC. For example, sarcopenia (loss of skeletal muscle mass and strength) and obesity predict poorer prognosis in CRC (6–8). Cancer is a catabolic disease characterized by muscle loss, often accompanied by fat gain, also known as less muscular obesity (9–11). This abnormal body composition phenotype occurs across the weight spectrum, with an 18% prevalence (12). Reduced skeletal muscle and increased adipose tissue may synergize to exacerbate body damage and metabolic disorders (13). Moreover, the metabolic profile associated with tumors preferentially affects subcutaneous adipose tissue, which accounts for 80% of the body’s adiposity (14–17). Therefore, it is important to elucidate its relationship with skeletal muscle.

Skeletal muscle area and subcutaneous fat area, measured by computed tomography (CT) at the level of the third lumbar vertebra (L3), are considered the most relevant areas for overall body composition and have become the gold standard for diagnosing sarcopenia (18, 19). These images can be easily obtained from the Medical Imaging Case system used for cancer diagnosis and prognostic follow-up, and reliable and accurate measurements can be made without increasing the burden and cost to the patient. To correlate skeletal muscle area and subcutaneous fat area with total muscle mass and total subcutaneous fat content, respectively, and to obtain relative measurements, they were normalized to the square of height to obtain the skeletal muscle index (SMI) and the subcutaneous fat index (SFI) (20). Although previous studies have shown an association between SMI and SFI and CRC, the evidence for an association between SMI and SFI based on prospective studies is very limited and it is not clear how causal the relationship is. Understanding the bidirectional relationship between SMI and SFI in CRC patients could provide valuable insights into the underlying mechanisms between muscle mass and obesity, as well as treatment and prognosis. The use of Random Intercept Cross-Lagged Panel Model (RI-CLPM) provides a unique opportunity to explore the temporal relationship between SMI and SFI in CRC patients. It allows the directionality of the relationship between the two variables to be examined after controlling for inter-individual confounders, leading to a more comprehensive understanding of their interactions (21).

This retrospective cohort study aimed to measure the SMI and SFI at the L3 vertebral segment by collecting perioperative CT images of CRC patients. The RI-CLPM was used to analyze the direction and strength of the longitudinal association between SMI and SFI at the individual level, while Cox regression was employed to examine the association between these indices and the prognosis of CRC patients, emphasizing their clinical importance. The findings may aid in developing tailored interventions targeting obesity and muscle mass in CRC patients, potentially improving patient outcomes.

## 2 Materials and methods

### 2.1 Study population

**Figure 1:**
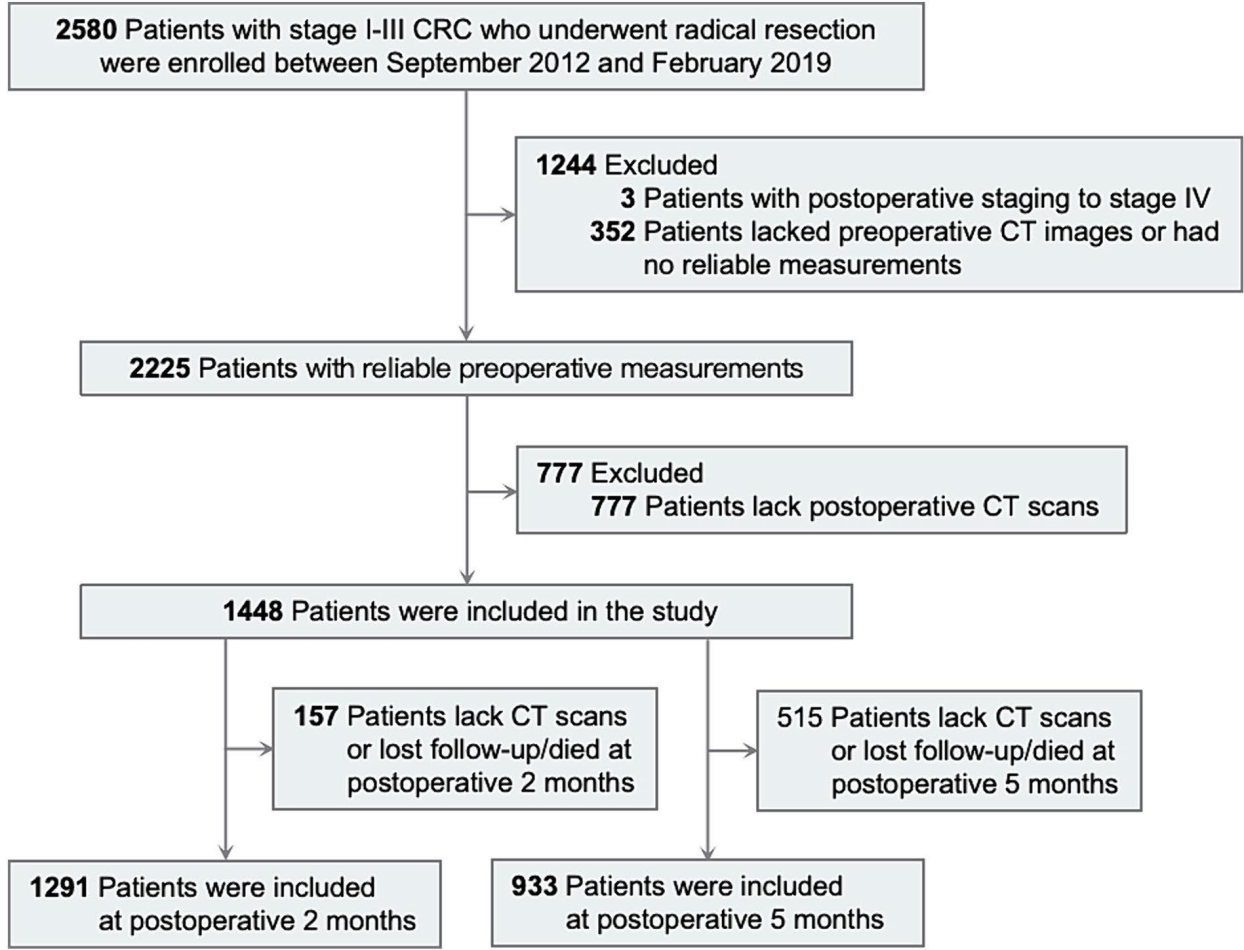
Study flow diagram.

The study protocol was approved by the Ethics Committee of Kunming Medical University. As this was a retrospective study, the Ethics Committee waived the requirement for informed consent. All data were anonymized. This study included consecutive patients with stage I-III primary colorectal cancer who underwent radical resection at the Third Affiliated Hospital of Kunming Medical University from September 2012 and February 2019 and met the inclusion and exclusion criteria. The hospital is a tertiary cancer care center serving Yunnan Province, China. All patients were pathologically confirmed. Exclusion criteria included postoperative staging to stage IV and the inability to obtain high-quality CT images during the perioperative period. CT scan images were retrospectively collected at three time points: preoperatively (P1), 2 months ± 60 days postoperatively (P2), and 5 months ± 60 days postoperatively (P3), for a total of 2,225 patients with reliable preoperative measurements. To ensure test power, only patients with complete preoperative data and at least one valid postoperative data point were included. The final cohort consisted of 1,448 patients, with the number reduced to 1,291 at 2 months postoperatively and 933 at 5 months postoperatively; the remaining data were used to assess lost to follow-up bias. The detailed inclusion and exclusion criteria are presented in Figure 1. This study followed the reporting guidelines of the Strengthening the Reporting of Observational Studies in Epidemiology (STROBE) (22).

### 2.2 CT examination

Abdominal contrast and non-contrast CT scans were performed using a CT scanner (SOMATOM Definition AS+, Siemens Healthineers, Germany). The abdominal CT images were obtained using a 128-row spiral CT scanner with a tube voltage of 120 kVp, tube current of 290–330 mA, and a rotation time of 0.50 s. The reconstruction image thickness for VP was 2.0 mm, and the image matrix was 512 × 512. Following this, the abdomen was radio scanned at a rate of 0.5 s per frame after an intravenous injection of Omnipaque GE Healthcare, USA (450 mg/kg) for approximately 70 s.

### 2.3 Image analysis

The radiologist identified the skeletal muscle and subcutaneous fat areas independently. One radiologist had 13 years of experience in abdominal imaging, while the other had 2 years of experience in abdominal imaging. The CT images from the study, whether enhanced or non-enhanced, were analyzed using Slice Omatic software (version 6.0), which is available on the open-source website https://www.slicer.org. The cross-sectional area of skeletal muscle and subcutaneous fat was measured at the level of the third lumbar vertebrae using a semi-automated method. The standard Hounsfield unit range (−190 to −30 for adipose tissue and −29 to +150 for skeletal muscle) was employed. Skeletal muscle included psoas, erector spine, lumbar square, transversus abdominis, external abdominal obliquity, internal abdominal obliquity, and rectus abdominis. The region of interest was adjusted manually to match the actual boundaries of the muscle and subcutaneous fat. The areas of skeletal muscle and subcutaneous fat were then calculated automatically. If the correlation coefficient between the measurements of the two radiologists was 0.90 or higher, the average of the two measurements was used as the result. If the correlation coefficient was lower than 0.90, a third radiologist (with over 25 years of clinical experience in CT scanning and image post-processing) measured the skeletal muscle area and subcutaneous fat area again. The measurements taken by the third radiologist were used as the final result. Subsequently, the skeletal muscle area and subcutaneous fat area were normalized and divided by the square of the height (m^2^) to calculate the SMI and SFI. For subsequent analysis, measurements from patients with two or more observations within each time period were averaged.

### 2.4 Definitions of low SMI and high SFI

To further analyze the association between SMI and SFI with CRC prognosis, we applied the same methodology as previous studies to determine the optimal cutoff values based on baseline data (23). The X-tile software (version 3.6.1) was employed to identify cutoff points for continuous variables from gender-specific stratifications (23, 24). This software identifies the cutoff that best separates patient outcomes by maximizing the difference in survival between groups. For each candidate cutoff point, we compared recurrence-free survival (RFS) between the low SMI and normal SMI groups, as well as between the normal SFI and high SFI groups, calculated the log-rank statistics. The cutoff point yielding the highest log-rank statistic, indicating the most significant in RFS, was selected to distinguish between different SMI and SFI groups. These optimal cutoff values were then used to categorize the perioperative SMI into low SMI and normal SMI groups, and the SFI into normal SFI and high SFI groups.

In this study, the optimal cutoff values determined were 43.8 cm^2^/m^2^ for men and 34.3 cm^2^/m^2^ for women for SMI, and 46.20 cm^2^/m^2^ for men and 64.0 cm^2^/m^2^ for women for SFI. These values were chosen because they provided the greatest separation in survival outcomes within our cohort, thus enhancing the predictive accuracy of the Cox regression models in evaluating colorectal cancer prognosis.

### 2.5 Covariates and endpoints

Covariates were obtained from the electronic medical record, including gender, age, weight, preoperative to first postoperative weight change, BMI, smoking history, drinking history, hypertension, diabetes, ECOG performance status, Charlson comorbidity index, primary site, AJCC pathological stage, AJCC T stage, AJCC N stage, tumor differentiation, histologic type, lymph node yield, lymphovascular invasion, perineural invasion, tumor deposit, and adjuvant chemotherapy.

All patients were assessed for survival using an electronic medical record system and regular telephone follow-up. The primary outcome was recurrence-free survival (RFS), defined as the time from the date of surgery to cancer recurrence, death, or the last follow-up date. The secondary outcome was overall survival (OS), defined as the time from surgery to death from any cause. Data for patients lost to follow-up were censored at the date of the last known contact.

### 2.6 Missing data

We compared demographic and study variables between participants who were included in the study and those who remained. The final study population and those who dropped out did not differ significantly in sex (*p* = 0.075), primary site (*p* = 0.070), pathologic stage (*p* = 0.060), and postoperative SMI and SFI at all time points. However, significant differences were found in age (*p* < 0.001) and preoperative SMI (*p* = 0.003). Older patients, lower preoperative SMI were more likely to be lost to follow-up, as detailed in Supplementary Table 1. Among the study subjects in the analysis, 10.84% lost visits between the first and second measurements, and 35.57% lost visits between the first and third measurements. Comparisons of clinical variables between the included participants and those lost to follow-up are detailed in Supplementary Tables 2, 3. We conducted the primary analysis of this study using full information maximum likelihood (FIML) estimation, an appropriate method for estimating a structural equation model when the data are missing at random or non-randomly (25). FIML estimation for non-MCAR data is preferable to other methods for dealing with missing values, such as deletion by list (26).

### 2.7 Statistical analysis

**Figure 2:**
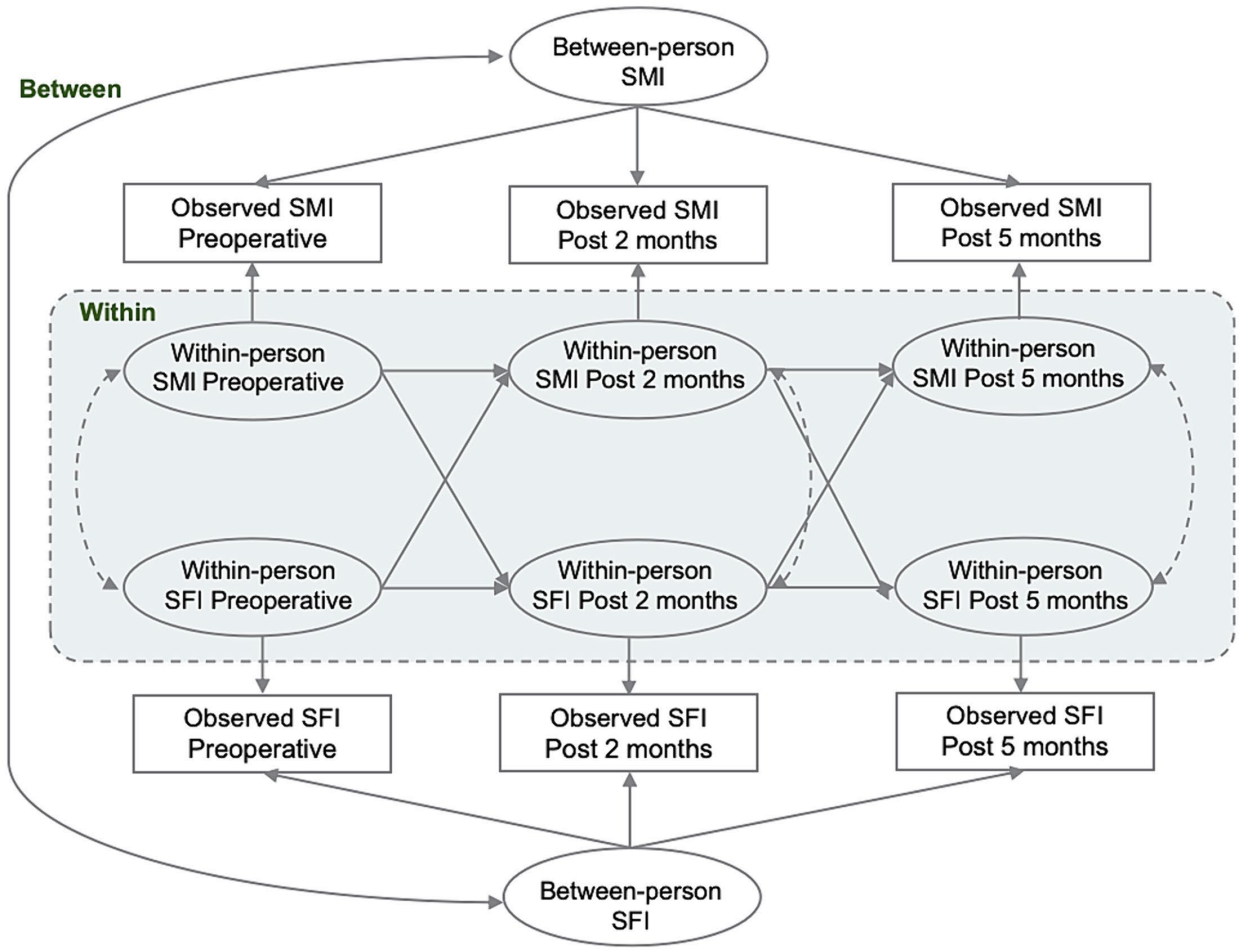
Conceptual model of the random-intercept cross-lagged panel analysis.

Continuous variables were presented as mean ± standard deviation or median [quartiles]. Group comparisons were made using either the paired t-test or the non-parametric rank-sum test. The study expressed categorical variables as frequencies (percentages) and compared them between groups using either the chi-square test or Fisher’s exact test. RI-CLPM was used to examine the within-person association of SMI and SFI at P1, P2, and P3. The complete RI-CLPM path diagram is presented in Figure 2. For each variable of interest, we conducted a regression analysis using the variables observed at P1, P2, and P3. The regression weights were constrained to be equal and were regressed on a time-invariant latent factor, the random intercept. This factor represents the stabilizing effect on the model over the observation period. Additionally, we regressed the variables on a separate latent factor at each time point, which represents the time-specific bias of the within-individual level at measurement. Cross-lagged and autoregressive coefficients were specified and freely estimated between the time-varying latent factors. These coefficients were interpreted as associations between within-person changes in SMI and SFI over the specified time interval. The within-person correlations were used to test the hypothesis that SMI and SFI would be prospectively correlated over time. Correlations with random intercepts represent the time-invariant effects of unmeasured sources of interindividual variance, such as sex and age, on the overall relationship between the constructs. Model fit was evaluated using chi-square (*χ*^2^), comparative goodness-of-fit indices, Tucker-Lewis indices, approximated root-mean-square errors, and standardized root-mean-square residuals. Standardized estimates were reported and compared for all analyses. In the survival analysis, univariate and multivariate Cox proportional hazards models were used to evaluate the crude and adjusted associations between perioperative SMI, SFI, their combination, and RFS and OS in CRC patients. Variables that achieved a less strict significance level of *p* < 0.10 in the univariate analysis were included in the subsequent multivariate model. Hazard ratios (HRs) and corresponding 95% confidence intervals (CIs) were estimated.

The data was cleaned and fitting of Cox proportional hazards models were conducted using the R statistical computing environment, version 4.3.0. A random intercept cross-lagged panel analysis was conducted using Mplus v.8.7, with a significance threshold of 0.05.

## 3 Results

### 3.1 Clinical and demographic variables by SMI and SFI

Clinical and demographic variables were compared based on SMI levels at P1, P2, and P3 (Table 1) and SFI levels at P1, P2, and P3 (Table 2). A total of 1,488 patients were included in the study. The median age at diagnosis was 59.00 years (IQR: 51.00–66.00), and 587 (40.54%) of the cases were female. At P1, 358 patients (24.72%) were classified as low SMI; at P2, 320 patients (24.79%) were low SMI; and at P3, 182 patients (19.51%) were low SMI. For SFI, 392 patients (27.07%) were classified as low SFI at P1; 282 patients (21.84%) at P2; and 224 patients (24.03%) at P3. Patients with low SMI at all three time points were older (*p* < 0.001) and had lower BMI (*p* < 0.001). High SFI patients were more likely to be female (*p* < 0.001) and had higher BMI (*p* < 0.001). At P1, low SMI was more prevalent in patients with colon cancer (*p* = 0.010), while at P3, it was more prevalent in patients with rectal cancer (*p* = 0.007). No significant differences were found across different pathological stages. At P1, high SFI was more common in patients with stage III cancer (*p* = 0.012), with no significant differences observed across primary sites.

**Table 1:** Demographic and clinical characteristics in colorectal cancer cohort by P1, P2, and P3 SMI.

Variable	P1 Skeletal muscle index			*p* value	P2 Skeletal muscle index			*p* value	P3 Skeletal muscle index			*p* value
	All (*n* = 1448)	Low group (*n* = 358)	Normal group (*n* = 1090)		All (*n* = 1291)	Low group (*n* = 320)	Normal group (*n* = 971)		All (*n* = 933)	Low group (*n* = 182)	Normal group (*n* = 751)	
Age (Median, IQR)	59.00 (51.00, 66.00)	64.00 (55.00, 71.00)	58.00 (50.00, 65.00)	<0.001	59.00 (50.00, 66.00)	62.00 (53.00, 69.25)	58.00 (50.00, 65.00)	<0.001	59.00 (50.00, 65.00)	64.00 (56.00, 71.00)	57.00 (49.00, 64.00)	<0.001
Sex, (*n*, %)				0.149				0.010				0.124
Female	587 (40.54)	133 (37.15)	454 (41.65)		525 (40.67)	110 (34.38)	415 (42.74)		367 (39.34)	62 (34.07)	305 (40.61)	
Male	861 (59.46)	225 (62.85)	636 (58.35)		766 (59.33)	210 (65.62)	556 (57.26)		566 (60.66)	120 (65.93)	446 (59.39)	
Weight (Median, IQR)	60.00 (54.00, 68.00)	61.00 (55.00, 70.00)	57.00 (51.62, 61.00)	<0.001	60.00 (54.00, 68.00)	61.00 (55.00, 70.00)	59.00 (52.00, 64.00)	<0.001	60.00 (55.00, 68.00)	61.00 (55.00, 69.00)	60.00 (53.00, 65.00)	<0.001
Weight change at first postoperative follow-up (Median, IQR)	−3.00 (−5.00, −1.00)	−3.00 (−5.00, −1.00)	−3.00 (−5.00, −1.00)	0.114	−3.00 (−5.00, −1.00)	−3.25 (−5.88, −2.00)	−3.00 (−5.00, −1.00)	0.002	−3.00 (−5.00, −1.00)	−3.00 (−6.00, −1.00)	−3.00 (−5.00, −1.00)	0.283
BMI (Median, IQR)	22.66 (20.76, 25.00)	20.76 (19.06, 22.30)	23.44 (21.33, 25.69)	<0.001	22.68 (20.76, 25.12)	20.88 (19.56, 22.68)	23.44 (21.23, 25.70)	<0.001	22.66 (20.76, 25.10)	20.99 (19.25, 22.58)	23.31 (21.09, 25.39)	<0.001
Smoking history (*n*, %)				0.962				0.166				0.866
Yes	380 (26.24)	94 (26.26)	286 (26.24)		349 (27.03)	95 (29.69)	254 (26.16)		246 (26.37)	45 (24.73)	201 (26.76)	
No	1,053 (72.72)	260 (72.63)	793 (72.75)		930 (72.04)	220 (68.75)	710 (73.12)		680 (72.88)	136 (74.73)	544 (72.44)	
Unknow	15 (1.04)	4 (1.12)	11 (1.01)		12 (0.93)	5 (1.56)	7 (0.72)		7 (0.75)	1 (0.55)	6 (0.80)	
Drinking history (*n*, %)				0.583				0.642				0.793
Yes	282 (19.48)	63 (17.60)	219 (20.09)		257 (19.91)	64 (20.00)	193 (19.88)		185 (19.83)	34 (18.68)	151 (20.11)	
No	1,108 (76.52)	280 (78.21)	828 (75.96)		981 (75.99)	240 (75.00)	741 (76.31)		717 (76.85)	143 (78.57)	574 (76.43)	
Unknow	58 (4.01)	15 (4.19)	43 (3.94)		53 (4.11)	16 (5.00)	37 (3.81)		31 (3.32)	5 (2.75)	26 (3.46)	
Hypertension, (*n*, %)				0.910				0.569				>0.999
Yes	343 (23.69)	87 (24.30)	256 (23.49)		305 (23.63)	69 (21.56)	236 (24.30)		218 (23.37)	42 (23.08)	176 (23.44)	
No	1,100 (75.97)	270 (75.42)	830 (76.15)		982 (76.07)	250 (78.12)	732 (75.39)		713 (76.42)	140 (76.92)	573 (76.30)	
Unknow	5 (0.35)	1 (0.28)	4 (0.37)		4 (0.31)	1 (0.31)	3 (0.31)		2 (0.21)	0 (0.00)	2 (0.27)	
Diabetes (*n*, %)				0.183				0.503				0.214
Yes	125 (8.63)	39 (10.89)	86 (7.89)		113 (8.75)	29 (9.06)	84 (8.65)		75 (8.04)	20 (10.99)	55 (7.32)	
No	1,315 (90.81)	317 (88.55)	998 (91.56)		1,171 (90.70)	288 (90.00)	883 (90.94)		855 (91.64)	162 (89.01)	693 (92.28)	
Unknow	8 (0.55)	2 (0.56)	6 (0.55)		7 (0.54)	3 (0.94)	4 (0.41)		3 (0.32)	0 (0.00)	3 (0.40)	
ECOG (*n*, %)				<0.001				0.031				0.979
0	796 (54.97)	192 (53.63)	604 (55.41)		719 (55.69)	172 (53.75)	547 (56.33)		508 (54.45)	98 (53.85)	410 (54.59)	
1	601 (41.51)	139 (38.83)	462 (42.39)		528 (40.90)	130 (40.62)	398 (40.99)		394 (42.23)	78 (42.86)	316 (42.08)	
2	22 (1.52)	13 (3.63)	9 (0.83)		16 (1.24)	9 (2.81)	7 (0.72)		14 (1.50)	3 (1.65)	11 (1.46)	
≥3	29 (2.00)	14 (3.91)	15 (1.38)		28 (2.17)	9 (2.81)	19 (1.96)		17 (1.82)	3 (1.65)	14 (1.86)	
Charlson comorbidity index (*n*, %)				<0.001				<0.001				<0.001
0	303 (20.93)	50 (13.97)	253 (23.21)		280 (21.69)	49 (15.31)	231 (23.79)		207 (22.19)	22 (12.09)	185 (24.63)	
1	391 (27.00)	75 (20.95)	316 (28.99)		346 (26.80)	74 (23.12)	272 (28.01)		262 (28.08)	36 (19.78)	226 (30.09)	
2	457 (31.56)	114 (31.84)	343 (31.47)		412 (31.91)	104 (32.50)	308 (31.72)		294 (31.51)	63 (34.62)	231 (30.76)	
3	226 (15.61)	93 (25.98)	133 (12.20)		193 (14.95)	75 (23.44)	118 (12.15)		133 (14.26)	44 (24.18)	89 (11.85)	
≥4	67 (4.63)	25 (6.98)	42 (3.85)		57 (4.42)	17 (5.31)	40 (4.12)		36 (3.86)	17 (9.34)	19 (2.53)	
Unknow	4 (0.28)	1 (0.28)	3 (0.28)		3 (0.23)	1 (0.31)	2 (0.21)		1 (0.11)	0 (0.00)	1 (0.13)	
Primary site, (*n*, %)				0.010				0.394				0.007
Colon	709 (48.96)	197 (55.03)	512 (46.97)		650 (50.35)	154 (48.12)	496 (51.08)		460 (49.30)	73 (40.11)	387 (51.53)	
Rectum	739 (51.04)	161 (44.97)	578 (53.03)		641 (49.65)	166 (51.88)	475 (48.92)		473 (50.70)	109 (59.89)	364 (48.47)	
Pathological stage (*n*, %)				0.895				0.632				0.493
I	318 (21.96)	81 (22.63)	237 (21.74)		257 (19.91)	65 (20.31)	192 (19.77)		167 (17.90)	38 (20.88)	129 (17.18)	
II	570 (39.36)	142 (39.66)	428 (39.27)		521 (40.36)	122 (38.12)	399 (41.09)		384 (41.16)	71 (39.01)	313 (41.68)	
III	560 (38.67)	135 (37.71)	425 (38.99)		513 (39.74)	133 (41.56)	380 (39.13)		382 (40.94)	73 (40.11)	309 (41.15)	
Tumor differentiation (*n*, %)				0.443				0.312				0.285
Well+ Moderate	976 (67.40)	241 (67.32)	735 (67.43)		874 (67.70)	226 (70.62)	648 (66.74)		623 (66.77)	125 (68.68)	498 (66.31)	
Poor	367 (25.35)	86 (24.02)	281 (25.78)		328 (25.41)	71 (22.19)	257 (26.47)		250 (26.80)	50 (27.47)	200 (26.63)	
Unknown	105 (7.25)	31 (8.66)	74 (6.79)		89 (6.89)	23 (7.19)	66 (6.80)		60 (6.43)	7 (3.85)	53 (7.06)	
Histologic type (*n*, %)				0.670				0.452				>0.999
Mucinous type	1,348 (93.09)	331 (92.46)	1,017 (93.30)		1,196 (92.64)	300 (93.75)	896 (92.28)		861 (92.28)	168 (92.31)	693 (92.28)	
Non–Mucinous type	100 (6.91)	27 (7.54)	73 (6.70)		95 (7.36)	20 (6.25)	75 (7.72)		72 (7.72)	14 (7.69)	58 (7.72)	
T stage (*n*, %)				0.987				0.145				0.935
T1	106 (7.32)	25 (6.98)	81 (7.43)		82 (6.35)	13 (4.06)	69 (7.11)		50 (5.36)	9 (4.95)	41 (5.46)	
T2	267 (18.44)	65 (18.16)	202 (18.53)		229 (17.74)	63 (19.69)	166 (17.10)		153 (16.40)	32 (17.58)	121 (16.11)	
T3	997 (68.85)	249 (69.55)	748 (68.62)		910 (70.49)	230 (71.88)	680 (70.03)		678 (72.67)	132 (72.53)	546 (72.70)	
T4	78 (5.39)	19 (5.31)	59 (5.41)		70 (5.42)	14 (4.38)	56 (5.77)		52 (5.57)	9 (4.95)	43 (5.73)	
N stage (*n*, %)				0.688				0.416				0.476
N0	881 (60.84)	221 (61.73)	660 (60.55)		771 (59.72)	185 (57.81)	586 (60.35)		547 (58.63)	107 (58.79)	440 (58.59)	
N1	416 (28.73)	104 (29.05)	312 (28.62)		383 (29.67)	104 (32.50)	279 (28.73)		282 (30.23)	59 (32.42)	223 (29.69)	
N2	151 (10.43)	33 (9.22)	118 (10.83)		137 (10.61)	31 (9.69)	106 (10.92)		104 (11.15)	16 (8.79)	88 (11.72)	
Lymph node yield (*n*, %)				0.331				0.591				0.269
<12	276 (19.06)	75 (20.95)	201 (18.44)		247 (19.13)	65 (20.31)	182 (18.74)		166 (17.79)	38 (20.88)	128 (17.04)	
≥12	1,172 (80.94)	283 (79.05)	889 (81.56)		1,044 (80.87)	255 (79.69)	789 (81.26)		767 (82.21)	144 (79.12)	623 (82.96)	
Lymph vascular invasion (*n*, %)				0.563				0.276				0.086
Yes	112 (7.73)	23 (6.42)	89 (8.17)		102 (7.90)	19 (5.94)	83 (8.55)		85 (9.11)	12 (6.59)	73 (9.72)	
No	214 (14.78)	54 (15.08)	160 (14.68)		194 (15.03)	46 (14.37)	148 (15.24)		132 (14.15)	19 (10.44)	113 (15.05)	
Unknown	1,122 (77.49)	281 (78.49)	841 (77.16)		995 (77.07)	255 (79.69)	740 (76.21)		716 (76.74)	151 (82.97)	565 (75.23)	
Perineural invasion (*n*, %)				0.385				0.270				0.085
Yes	32 (2.21)	5 (1.40)	27 (2.48)		30 (2.32)	7 (2.19)	23 (2.37)		18 (1.93)	2 (1.10)	16 (2.13)	
No	274 (18.92)	64 (17.88)	210 (19.27)		249 (19.29)	52 (16.25)	197 (20.29)		183 (19.61)	26 (14.29)	157 (20.91)	
Unknown	1,142 (78.87)	289 (80.73)	853 (78.26)		1,012 (78.39)	261 (81.56)	751 (77.34)		732 (78.46)	154 (84.62)	578 (76.96)	
Tumor deposit (*n*, %)				0.892				0.948				0.382
Yes	165 (11.40)	42 (11.73)	123 (11.28)		154 (11.93)	39 (12.19)	115 (11.84)		118 (12.65)	19 (10.44)	99 (13.18)	
No	1,283 (88.60)	316 (88.27)	967 (88.72)		1,137 (88.07)	281 (87.81)	856 (88.16)		815 (87.35)	163 (89.56)	652 (86.82)	
Adjuvant chemotherapy, (*n*, %)				0.180				0.397				0.266
Yes	982 (67.82)	232 (64.80)	750 (68.81)		918 (71.11)	234 (73.12)	684 (70.44)		694 (74.38)	129 (70.88)	565 (75.23)	
No	466 (32.18)	126 (35.20)	340 (31.19)		373 (28.89)	86 (26.88)	287 (29.56)		239 (25.62)	53 (29.12)	186 (24.77)	
SMI Preoperative (Median, IQR)	44.13 (38.65, 49.76)	37.10 (32.54, 41.04)	46.84 (41.66, 51.56)	<0.001	44.19 (38.73, 49.94)	38.86 (33.99, 42.61)	46.49 (40.61, 51.70)	<0.001	44.43 (38.65, 50.03)	37.92 (33.52, 41.79)	46.02 (40.46, 51.29)	<0.001
SMI Post 2 months (Median, IQR)	44.15 (38.69, 49.51)	38.60 (34.09, 41.85)	46.28 (41.02, 51.23)	<0.001	44.15 (38.69, 49.51)	38.53 (32.94, 41.49)	46.83 (41.33, 51.28)	<0.001	44.47 (38.83, 49.86)	38.18 (32.95, 40.83)	46.28 (41.21, 51.26)	<0.001
SMI Post 5 months (Median, IQR)	45.61 (40.03, 51.51)	40.06 (35.24, 43.79)	47.93 (42.17, 53.18)	<0.001	45.74 (40.00, 51.70)	39.78 (34.09, 43.56)	48.59 (42.43, 53.37)	<0.001	45.61 (40.03, 51.51)	38.51 (33.19, 41.66)	47.76 (42.63, 52.95)	<0.001
SFI Preoperative (Median, IQR)	40.13 (27.95, 57.81)	32.55 (21.62, 46.80)	42.56 (30.14, 60.87)	<0.001	40.21 (28.30, 58.58)	33.53 (22.50, 47.20)	43.00 (29.76, 61.22)	<0.001	40.16 (28.62, 57.44)	34.06 (24.49, 50.67)	41.18 (29.69, 58.61)	<0.001
SFI Post 2 months (Median, IQR)	36.83 (25.36, 53.06)	30.42 (20.64, 43.28)	39.69 (27.24, 56.16)	<0.001	36.83 (25.36, 53.06)	29.09 (20.08, 40.33)	40.29 (27.41, 56.50)	<0.001	36.75 (25.49, 51.83)	31.83 (21.05, 46.14)	38.02 (27.12, 53.21)	<0.001
SFI Post 5 months (Median, IQR)	38.74 (27.63, 53.88)	33.28 (23.16, 42.93)	40.47 (29.74, 56.47)	<0.001	38.78 (27.53, 53.81)	31.84 (22.65, 42.30)	40.98 (29.88, 56.58)	<0.001	38.74 (27.63, 53.88)	31.33 (22.69, 43.36)	40.39 (29.75, 56.27)	<0.001

IQR, interquartile range; BMI, Body Mass Index; ECOG, eastern cooperative oncology group; SMI, skeletal muscle index; SFI, subcutaneous fat index; Post, postoperative.

**Table 2:** Demographic and clinical characteristics in colorectal cancer cohort by P1, P2, and P3 SFI.

Variable	P1 Subcutaneous fat index			*p* value	P2 Subcutaneous fat index			*p* value	P3 Subcutaneous fat index			*p* value
	All (*n* = 1448)	Normal group (*n* = 1057)	High group (*n* = 391)		All (*n* = 1291)	Normal group (*n* = 1012)	High group (*n* = 279)		All (*n* = 932)	Normal group (*n* = 708)	High group (*n* = 224)	
Age (Median, IQR)	59.00 (51.00, 66.00)	59.00 (51.00, 66.00)	60.00 (51.00, 67.00)	0.338	60.00 (54.00, 68.00)	60.00 (53.00, 66.00)	66.00 (59.50, 75.00)	<0.001	59.00 (50.00, 65.00)	59.00 (50.00, 65.00)	57.50 (50.00, 65.00)	0.894
Sex (*n*, %)				<0.001				<0.001				<0.001
Female	587 (40.54)	345 (32.64)	242 (61.89)		525 (40.67)	352 (34.78)	173 (62.01)		367 (39.38)	241 (34.04)	126 (56.25)	
Male	861 (59.46)	712 (67.36)	149 (38.11)		766 (59.33)	660 (65.22)	106 (37.99)		565 (60.62)	467 (65.96)	98 (43.75)	
Weight (Median, IQR)	60.00 (54.00, 68.00)	61.00 (55.00, 70.00)	57.00 (51.62, 61.00)	<0.001	59.00 (50.00, 66.00)	59.00 (50.00, 66.00)	59.00 (50.00, 66.00)	0.981	60.00 (55.00, 68.00)	60.00 (53.00, 66.00)	65.00 (60.00, 74.25)	<0.001
Weight change at first postoperative follow-up (Median, IQR)	−3.00 (−5.00, −1.00)	−3.00 (−5.00, −1.00)	−3.50 (−6.00, −1.00)	0.001	−3.00 (−5.00, −1.00)	−3.00 (−5.00, −1.00)	−3.00 (−6.00, −1.00)	0.667	−3.00 (−5.00, −1.00)	−3.00 (−5.00, −1.00)	−3.00 (−5.00, −1.00)	0.938
BMI (Median, IQR)	22.66 (20.76, 25.00)	21.78 (20.31, 23.62)	25.57 (23.77, 27.55)	<0.001	22.68 (20.76, 25.12)	22.04 (20.57, 24.03)	25.81 (23.88, 27.91)	<0.001	22.63 (20.76, 25.10)	21.98 (20.57, 24.04)	25.50 (23.24, 27.69)	<0.001
Smoking history (*n*, %)				<0.001				<0.001				<0.001
Yes	380 (26.24)	328 (31.03)	52 (13.30)		349 (27.03)	310 (30.63)	39 (13.98)		246 (26.39)	209 (29.52)	37 (16.52)	
No	1,053 (72.72)	718 (67.93)	335 (85.68)		930 (72.04)	693 (68.48)	237 (84.95)		679 (72.85)	494 (69.77)	185 (82.59)	
Unknow	15 (1.04)	11 (1.04)	4 (1.02)		12 (0.93)	9 (0.89)	3 (1.08)		7 (0.75)	5 (0.71)	2 (0.89)	
Drinking history (*n*, %)				<0.001				<0.001				0.053
Yes	282 (19.48)	245 (23.18)	37 (9.46)		257 (19.91)	224 (22.13)	33 (11.83)		184 (19.74)	149 (21.05)	35 (15.62)	
No	1,108 (76.52)	768 (72.66)	340 (86.96)		981 (75.99)	742 (73.32)	239 (85.66)		717 (76.93)	532 (75.14)	185 (82.59)	
Unknow	58 (4.01)	44 (4.16)	14 (3.58)		53 (4.11)	46 (4.55)	7 (2.51)		31 (3.33)	27 (3.81)	4 (1.79)	
Hypertension (*n*, %)				<0.001				<0.001				<0.001
Yes	343 (23.69)	208 (19.68)	135 (34.53)		305 (23.63)	206 (20.36)	99 (35.48)		218 (23.39)	140 (19.77)	78 (34.82)	
No	1,100 (75.97)	845 (79.94)	255 (65.22)		982 (76.07)	803 (79.35)	179 (64.16)		712 (76.39)	566 (79.94)	146 (65.18)	
Unknow	5 (0.35)	4 (0.38)	1 (0.26)		4 (0.31)	3 (0.30)	1 (0.36)		2 (0.21)	2 (0.28)	0 (0.00)	
Diabetes (*n*, %)				0.774				0.934				0.192
Yes	125 (8.63)	90 (8.51)	35 (8.95)		113 (8.75)	90 (8.89)	23 (8.24)		75 (8.05)	51 (7.20)	24 (10.71)	
No	1,315 (90.81)	960 (90.82)	355 (90.79)		1,171 (90.70)	916 (90.51)	255 (91.40)		854 (91.63)	654 (92.37)	200 (89.29)	
Unknow	8 (0.55)	7 (0.66)	1 (0.26)		7 (0.54)	6 (0.59)	1 (0.36)		3 (0.32)	3 (0.42)	0 (0.00)	
ECOG (*n*, %)				0.415				0.968				0.628
0	796 (54.97)	575 (54.40)	221 (56.52)		719 (55.69)	565 (55.83)	154 (55.20)		508 (54.51)	392 (55.37)	116 (51.79)	
1	601 (41.51)	444 (42.01)	157 (40.15)		528 (40.90)	413 (40.81)	115 (41.22)		393 (42.17)	291 (41.10)	102 (45.54)	
2	22 (1.52)	14 (1.32)	8 (2.05)		16 (1.24)	12 (1.19)	4 (1.43)		14 (1.50)	12 (1.69)	2 (0.89)	
≥3	29 (2.00)	24 (2.27)	5 (1.28)		28 (2.17)	22 (2.17)	6 (2.15)		17 (1.82)	13 (1.84)	4 (1.79)	
Charlson comorbidity index (*n*, %)				0.813				0.922				0.951
0	303 (20.93)	221 (20.91)	82 (20.97)		280 (21.69)	219 (21.64)	61 (21.86)		207 (22.21)	159 (22.46)	48 (21.43)	
1	391 (27.00)	295 (27.91)	96 (24.55)		346 (26.80)	274 (27.08)	72 (25.81)		262 (28.11)	195 (27.54)	67 (29.91)	
2	457 (31.56)	330 (31.22)	127 (32.48)		412 (31.91)	320 (31.62)	92 (32.97)		293 (31.44)	224 (31.64)	69 (30.80)	
3	226 (15.61)	159 (15.04)	67 (17.14)		193 (14.95)	150 (14.82)	43 (15.41)		133 (14.27)	100 (14.12)	33 (14.73)	
≥4	67 (4.63)	49 (4.64)	18 (4.60)		57 (4.42)	47 (4.64)	10 (3.58)		36 (3.86)	29 (4.10)	7 (3.12)	
Unknow	4 (0.28)	3 (0.28)	1 (0.26)		3 (0.23)	2 (0.20)	1 (0.36)		1 (0.11)	1 (0.14)	0 (0.00)	
Primary site (*n*, %)				0.901				0.895				0.127
Colon	709 (48.96)	516 (48.82)	193 (49.36)		650 (50.35)	511 (50.49)	139 (49.82)		460 (49.36)	339 (47.88)	121 (54.02)	
Rectum	739 (51.04)	541 (51.18)	198 (50.64)		641 (49.65)	501 (49.51)	140 (50.18)		472 (50.64)	369 (52.12)	103 (45.98)	
Pathological stage (*n*, %)				0.014				0.345				0.510
I	318 (21.96)	226 (21.38)	92 (23.53)		257 (19.91)	194 (19.17)	63 (22.58)		167 (17.92)	123 (17.37)	44 (19.64)	
II	570 (39.36)	440 (41.63)	130 (33.25)		521 (40.36)	417 (41.21)	104 (37.28)		383 (41.09)	298 (42.09)	85 (37.95)	
III	560 (38.67)	391 (36.99)	169 (43.22)		513 (39.74)	401 (39.62)	112 (40.14)		382 (40.99)	287 (40.54)	95 (42.41)	
Tumor differentiation (*n*, %)				0.771				0.391				0.200
Well+ Moderate	976 (67.40)	707 (66.89)	269 (68.80)		874 (67.70)	676 (66.80)	198 (70.97)		622 (66.74)	462 (65.25)	160 (71.43)	
Poor	367 (25.35)	273 (25.83)	94 (24.04)		328 (25.41)	263 (25.99)	65 (23.30)		250 (26.82)	200 (28.25)	50 (22.32)	
Unknown	105 (7.25)	77 (7.28)	28 (7.16)		89 (6.89)	73 (7.21)	16 (5.73)		60 (6.44)	46 (6.50)	14 (6.25)	
Histologic type (*n*, %)				0.907				0.790				0.731
Mucinous type	1,348 (93.09)	983 (93.00)	365 (93.35)		1,196 (92.64)	936 (92.49)	260 (93.19)		860 (92.27)	655 (92.51)	205 (91.52)	
Non–Mucinous type	100 (6.91)	74 (7.00)	26 (6.65)		95 (7.36)	76 (7.51)	19 (6.81)		72 (7.73)	53 (7.49)	19 (8.48)	
T stage (*n*, %)				0.411				0.797				0.855
T1	106 (7.32)	77 (7.28)	29 (7.42)		82 (6.35)	63 (6.23)	19 (6.81)		50 (5.36)	37 (5.23)	13 (5.80)	
T2	267 (18.44)	185 (17.50)	82 (20.97)		229 (17.74)	176 (17.39)	53 (19.00)		153 (16.42)	116 (16.38)	37 (16.52)	
T3	997 (68.85)	740 (70.01)	257 (65.73)		910 (70.49)	720 (71.15)	190 (68.10)		677 (72.64)	513 (72.46)	164 (73.21)	
T4	78 (5.39)	55 (5.20)	23 (5.88)		70 (5.42)	53 (5.24)	17 (6.09)		52 (5.58)	42 (5.93)	10 (4.46)	
N stage (*n*, %)				0.083				0.606				0.739
N0	881 (60.84)	661 (62.54)	220 (56.27)		771 (59.72)	606 (59.88)	165 (59.14)		546 (58.58)	417 (58.90)	129 (57.59)	
N1	416 (28.73)	293 (27.72)	123 (31.46)		383 (29.67)	295 (29.15)	88 (31.54)		282 (30.26)	210 (29.66)	72 (32.14)	
N2	151 (10.43)	103 (9.74)	48 (12.28)		137 (10.61)	111 (10.97)	26 (9.32)		104 (11.16)	81 (11.44)	23 (10.27)	
Lymph node yield (*n*, %)				0.176				0.479				0.602
<12	276 (19.06)	192 (18.16)	84 (21.48)		247 (19.13)	189 (18.68)	58 (20.79)		166 (17.81)	123 (17.37)	43 (19.20)	
≥12	1,172 (80.94)	865 (81.84)	307 (78.52)		1,044 (80.87)	823 (81.32)	221 (79.21)		766 (82.19)	585 (82.63)	181 (80.80)	
Lymph vascular invasion (*n*, %)				0.389				0.604				0.881
Yes	112 (7.73)	83 (7.85)	29 (7.42)		102 (7.90)	77 (7.61)	25 (8.96)		85 (9.12)	65 (9.18)	20 (8.93)	
No	214 (14.78)	148 (14.00)	66 (16.88)		194 (15.03)	149 (14.72)	45 (16.13)		132 (14.16)	98 (13.84)	34 (15.18)	
Unknown	1,122 (77.49)	826 (78.15)	296 (75.70)		995 (77.07)	786 (77.67)	209 (74.91)		715 (76.72)	545 (76.98)	170 (75.89)	
Perineural invasion (*n*, %)				0.548				0.426				0.132
Yes	32 (2.21)	22 (2.08)	10 (2.56)		30 (2.32)	22 (2.17)	8 (2.87)		18 (1.93)	10 (1.41)	8 (3.57)	
No	274 (18.92)	194 (18.35)	80 (20.46)		249 (19.29)	189 (18.68)	60 (21.51)		183 (19.64)	139 (19.63)	44 (19.64)	
Unknown	1,142 (78.87)	841 (79.56)	301 (76.98)		1,012 (78.39)	801 (79.15)	211 (75.63)		731 (78.43)	559 (78.95)	172 (76.79)	
Tumor deposit (*n*, %)				0.268				0.276				0.340
Yes	165 (11.40)	114 (10.79)	51 (13.04)		154 (11.93)	115 (11.36)	39 (13.98)		118 (12.66)	85 (12.01)	33 (14.73)	
No	1,283 (88.60)	943 (89.21)	340 (86.96)		1,137 (88.07)	897 (88.64)	240 (86.02)		814 (87.34)	623 (87.99)	191 (85.27)	
Adjuvant chemotherapy (*n*, %)				0.499				0.778				0.157
Yes	982 (67.82)	711 (67.27)	271 (69.31)		918 (71.11)	722 (71.34)	196 (70.25)		693 (74.36)	535 (75.56)	158 (70.54)	
No	466 (32.18)	346 (32.73)	120 (30.69)		373 (28.89)	290 (28.66)	83 (29.75)		239 (25.64)	173 (24.44)	66 (29.46)	
SMI Preoperative (Median, IQR)	44.13 (38.65, 49.76)	43.97 (38.30, 49.68)	44.66 (39.22, 50.14)	0.191	44.19 (38.73, 49.94)	43.95 (38.24, 49.74)	45.22 (39.95, 50.35)	0.034	44.42 (38.65, 49.98)	44.19 (38.27, 49.69)	45.06 (39.36, 50.47)	0.113
SMI Post 2 months (Median, IQR)	44.15 (38.69, 49.51)	44.22 (38.63, 49.35)	43.97 (38.84, 49.69)	0.625	44.15 (38.69, 49.51)	44.01 (38.44, 49.28)	45.25 (40.02, 50.27)	0.009	44.49 (38.83, 49.87)	44.23 (38.66, 49.53)	45.43 (39.67, 51.05)	0.063
SMI Post 5 months (Median, IQR)	45.61 (40.03, 51.51)	45.36 (39.89, 51.64)	46.27 (40.54, 51.37)	0.432	45.74 (40.00, 51.70)	45.51 (39.64, 51.70)	46.47 (41.38, 51.57)	0.262	45.61 (40.03, 51.52)	45.28 (39.34, 51.29)	46.68 (41.04, 53.15)	0.017
SFI Preoperative (Median, IQR)	40.13 (27.95, 57.81)	33.28 (22.82, 42.78)	69.24 (58.11, 80.40)	<0.001	40.21 (28.30, 58.58)	34.72 (24.04, 46.00)	71.24 (60.53, 84.35)	<0.001	40.12 (28.62, 57.23)	34.68 (24.96, 45.42)	65.12 (53.46, 80.13)	<0.001
SFI Post 2 months (Median, IQR)	36.83 (25.36, 53.06)	30.42 (21.36, 40.30)	64.27 (51.82, 76.75)	<0.001	36.83 (25.36, 53.06)	31.97 (22.19, 42.00)	69.12 (57.81, 79.92)	<0.001	36.74 (25.48, 51.81)	31.72 (22.21, 41.09)	62.93 (51.65, 78.16)	<0.001
SFI Post 5 months (Median, IQR)	38.74 (27.63, 53.88)	33.17 (24.38, 41.95)	63.28 (51.32, 77.26)	<0.001	38.78 (27.53, 53.81)	33.92 (24.84, 43.22)	71.10 (56.91, 81.83)	<0.001	38.74 (27.63, 53.88)	33.38 (24.84, 41.57)	68.94 (54.95, 78.52)	<0.001

IQR, interquartile range; BMI, Body Mass Index; ECOG, eastern cooperative oncology group; SMI, skeletal muscle index; SFI, subcutaneous fat index; Post, postoperative.

### 3.2 Clinical and demographic variables by overall survival status and recurrence-free survival status

Clinical and demographic variables by survival status and relapse-free survival status are presented in Supplementary Table 4. The median follow-up time for the entire cohort was 51.47 months (IQR: 50.10–52.67). During the follow-up period, we observed 185 deaths and 375 recurrences. Patients who died or experienced recurrence were more likely to have stage III disease, higher pathological differentiation, and to have received adjuvant chemotherapy, *p* < 0.05. No significant differences were found in age, gender, or BMI, *p* > 0.05.

### 3.3 Bidirectional associations of SMI and SFI

**Figure 3:**
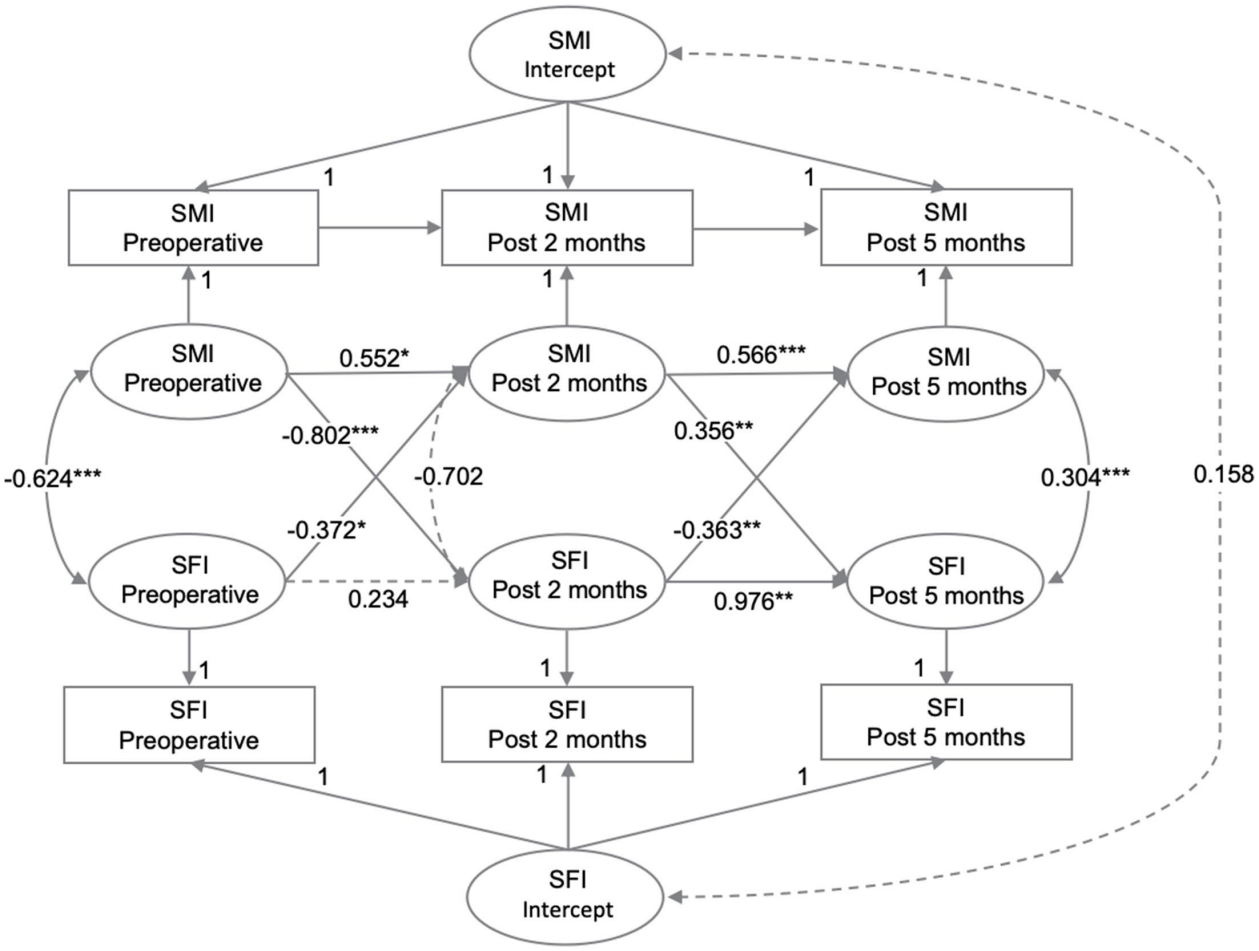
The relationships between SMI and SFI were analyzed at preoperative, post 2 months, and post 5 months using RI-CLPM. Solid black arrows indicate significant regression weights or correlations, while dashed arrows indicate non-significant parameters (*p* > 0.05). Standardized estimates are provided.

The intra-individual associations between SMI and SFI at three time points were analyzed using RI-CLPM. The regression coefficients for each path in the model are shown in Figure 3. At the individual level, SMI at P1 negatively predicted SFI at P2 (*β* = −0.802, *p* < 0.001). Similarly, SFI at P1 negatively predicted SMI at P2 (*β* = −0.372, *p* = 0.040). However, SMI at P2 positively predicted SFI at P3 (*β* = 0.356, *p* = 0.006), and SFI at P2 negatively predicted SFI at P3 (*β* = −0.363, *p* = 0.001). At P2-P3, the influence of subcutaneous fat on skeletal muscle and vice versa was reduced compared to P1-P2. Additionally, the negative effect of subcutaneous fat on skeletal muscle was greater than the positive effect of skeletal muscle on subcutaneous fat (*p* = 0.001). As a sensitivity analysis, the model was re-run using data with complete measurements at all three time points. Given the potential differences in disease progression and nutritional status between colon and rectal cancer patients, we adjusted for the primary tumor site in the model, yielding similar conclusions, as shown in Supplementary Figure 1.

The autoregressive effect of SMI was significant at both P1-P2 (*β* = 0.552, *p* = 0.019) and P2-P3 (*β* = 0.566, *p* < 0.001), indicating that SMI was prospectively associated with itself at all time points. In contrast, SFI at P1 was not associated with SFI at P2 (*β* = 0.234, *p* = 0.082), although SFI at P2 predicted SFI at P3 (*β* = 0.976, *p* = 0.001). There was no significant correlation between the random intercepts for SFI and SMI at the person-to-person level (*r* = 0.158, *p* = 0.604).

The model fit indices indicated a very good fit: (χ^2^/df (1) = 8.524, *p* = 0.004, CFI = 0.999, RMSEA = 0.072 [90% CI: 0.033–0.120]), which met or exceeded the traditional thresholds of χ^2^/df < 2, CFI > 0.95, and RMSEA <0.08 (27, 28). However, our model had a low degree of freedom (df = 1), which could indicate an overly stringent estimation of model fit (29). Therefore, we relied mainly on χ^2^/df and CFI to assess model fit.

### 3.4 Survival analysis

In the univariate and multivariable Cox regression analyses, seven potential covariates were identified in the univariate model: ECOG, pathological stage, tumor differentiation, lymphovascular invasion, perineural invasion, tumor deposits, and adjuvant chemotherapy (Supplementary Tables 5, 6). After adjusting for these factors in the multivariable model, At P1, the group with elevated SFI exhibited worse recurrence-free survival compared to the group with normal SFI (adjusted hazard ratio [aHR] = 1.24, 95% CI: 1.00–1.55). However, no significant associations were found between SFI at P2 and P3, SMI at P1-P3, and colorectal cancer prognosis. Furthermore, we examined the combined effects of low SMI and high SFI on prognosis. We observed that patients with either preoperative low SMI or preoperative high SFI had poorer recurrence-free survival (aHR = 1.39, 95% CI: 1.04–1.87) and overall survival (aHR = 1.26, 95% CI: 1.03–1.55) compared to patients with normal preoperative SMI and SFI. No similar associations were observed postoperatively (Supplementary Tables 7, 8).

## 4 Discussion

This study investigated the bidirectional causal relationship between perioperative SMI and SFI in CRC patients, and validated their association with CRC prognosis. The within-person analyses supported a bidirectional relationship between SMI and SFI, with varying patterns over time. Our model indicated that SMI at P1 negatively predicted SFI at P2, while SMI at P2 positively predicted SFI at P3, but this effect diminished at later time intervals. Meanwhile, SFI negatively predicted SMI at both P2 and P3, with relatively large effect sizes during P2-P3. Therefore, the predominant direction of the effect was SMI- > SFI during P1-P2, but SFI- > SMI during P2-P3. Additionally, multivariable Cox regression analysis revealed that patients with elevated preoperative SFI had poorer recurrence-free survival. Further analysis found that compared to patients with normal preoperative SMI and SFI, those with low SMI or high SFI had worse recurrence-free survival and overall survival.

This model may reflect perioperative body composition changes in CRC patients. Previous longitudinal studies have shown that most patients undergoing resection for CRC lose skeletal muscle and subcutaneous fat due to impaired food digestion and lower physical activity. In contrast, in the postoperative period, patients with I-III stage CRC gradually restore their body function and metabolic homeostasis, which decreases the breakdown and release of subcutaneous adipose tissue and increases the synthesis and growth of skeletal muscle tissue (30), as also supported by these data. The larger effect sizes from P1 to P2 for the SMI- > SFI relationship suggest a direct and/or specific relationship between the two. Skeletal muscle is the largest energy-consuming organ in the body, and its metabolic activity and function are related to energy balance and fat oxidation (31, 32). The physiology and morphology of skeletal muscle change with age, with skeletal muscle mass and strength declining linearly from the fourth decade of life, and up to 50% loss of skeletal muscle mass by the eighth decade of life (33). Loss of skeletal muscle leads to a 4% per decade decline in basal metabolic rate after age 50 (34, 35). Simultaneously, the lower physical activity following muscle loss reduces total energy expenditure and causes fat accumulation (36). Moreover, skeletal muscle, as the primary site of insulin-stimulated glucose uptake, has been implicated as a major driver of systemic insulin resistance; patients with skeletal muscle atrophy often develop insulin resistance, resulting in lower glucose utilization, which in turn stimulates fat synthesis and storage (37).

The larger effect size of SFI- > SMI from P2-P3 may imply that the subcutaneous fat increase due to skeletal muscle loss may feedback-regulate skeletal muscle changes, causing further skeletal muscle reduction in a vicious cycle. Previous studies have demonstrated that obesity causes a persistent low-grade inflammation in the body and the production of pro-inflammatory factors, such as TNF-*α*, IL-1β, IL-6, etc., which inhibit skeletal muscle cell proliferation and differentiation, promote skeletal muscle protein degradation, and induce apoptosis, reducing skeletal muscle mass through various signaling pathways (38, 39). Moreover, adipokine, secreted by adipose tissue, regulates insulin resistance and metabolic homeostasis (40), and subcutaneous adipose tissue increase releases more leptin (the adipokine prototype) (41), It decreases myofibrillar protein synthesis in skeletal muscle without changing circulating insulin levels, which then inhibits skeletal muscle tissue synthesis and growth (42). These metabolic disorders are intertwined in a vicious recurrent cycle of skeletal muscle mass reduction and adiposity (obesity) increase, resulting in “sarcopenic obesity” (43, 44).

Our results agree with Kim TN and colleagues’ findings, who reported that baseline visceral fat area (VFA) measured by CT negatively predicted changes in appendicular lean soft tissue (ALST) mass calculated using dual-energy X-ray absorptiometry, but baseline ALST mass did not predict changes in VFA during follow-up (45). Our results indicated that preoperative SMI negatively predicted SFI at P2, but SMI at P2 positively predicted SFI at P3, which may partly account for why baseline muscle mass did not predict fat area changes during follow-up. Moreover, Kim TN and colleagues’ study had only 379 participants, and they stressed that a larger sample size may show that baseline ALST mass is an independent risk factor for visceral obesity development (45). Therefore, there is increasing evidence that age-related skeletal muscle loss increases adipose tissue accumulation in the subcutis, which then causes further skeletal muscle loss.

Additionally, the results indicated that SMI at P2 positively influenced SFI at P3, possibly reflecting overall health and physical recovery during the postoperative period. Early postoperative increases in skeletal muscle mass are often accompanied by increased nutrient intake, which not only promotes muscle recovery but may also lead to increased subcutaneous fat. Our findings showed that SMI increased from 44.15 (38.69, 49.51) at P2 to 45.61 (40.03, 51.51) at P3, while SFI increased from 36.83 (25.36, 53.06) at P2 to 38.74 (27.63, 53.88) at P3. Although increased skeletal muscle mass raises metabolic rate and promotes fat consumption, the overall improvement in nutritional status and physical recovery may result in a net increase in fat stores. Conversely, SFI at P2 negatively affected SMI at P3, potentially due to the inhibitory effect of subcutaneous fat accumulation on muscle growth. During the postoperative recovery period, increased fat may be associated with inflammatory responses and metabolic stress, which can hinder muscle growth (46, 47).

Our study demonstrates that colorectal cancer patients with elevated preoperative subcutaneous fat index (SFI) have significantly lower recurrence-free survival rates compared to those with normal preoperative SFI. Furthermore, combined analysis reveals that patients with either low preoperative skeletal muscle index (SMI) or high preoperative SFI exhibit reduced overall and recurrence-free survival. These findings are consistent with existing literature, underscoring the importance of body composition in colorectal cancer prognosis. High SFI is associated with poorer outcomes potentially due to the promotion of inflammation and immune suppression in the tumor microenvironment, which accelerates tumor progression. Previous studies indicate that elevated SFI can affect tumor behavior and patient prognosis through mechanisms involving cytokines and growth factors secreted by adipose tissue (48, 49). Additionally, low SMI, indicative of muscle loss or atrophy, is often linked to frailty and poor survival outcomes. Research suggests that decreased skeletal muscle may lead to diminished immune function and reduced treatment tolerance, thereby impacting overall and recurrence-free survival (50–52). Although postoperative SFI and SMI measurements did not show significant associations, this could be attributed to the complexity of body composition changes post-surgery and the effects of surgical and therapeutic interventions. The variability during postoperative recovery may obscure the associations observed preoperatively. Furthermore, preoperative body composition indicators likely better reflect baseline health status and physiological reserves, thus providing a more accurate prediction of prognosis (53).

This study has several strengths. First, the authors used RI-CLPM to investigate the bidirectional association between SMI and SFI, which is believed to be the first study to do so. Second, we further validated the predictive value of SFI, SMI, and their combination for colorectal cancer prognosis. The results suggest that increasing preoperative skeletal muscle levels can prevent subcutaneous fat accumulation, thereby maintaining physical health and improving prognosis in colorectal cancer patients. This finding is significant for understanding and addressing changes and imbalances in body composition among colorectal cancer patients and for its clinical relevance. However, the study has certain limitations. Firstly, being a single-center retrospective study, its generalizability might be limited, potentially introducing bias. Secondly, due to the loss of cancer patients during follow-up, long-term effective CT images and measurement results could not be obtained. Only patients with more than two measurements from preoperative to 6 months postoperative were included to ensure a sufficient sample size, which might introduce bias and overlook long-term effects over time. Thirdly, the study variables SMI and SFI measured at three time points varied to different extents by gender, age, tumor primary site, and pathological stage. Due to the limited sample size, further stratified analysis could not be performed. Future studies might examine if similar effects exist in different populations.

Moreover, although we controlled for variance components such as gender, age, and pathological type before measurement, potential confounding factors such as tumor treatment, nutritional intake, perioperative physical activity, and resistance training could not be stabilized or predicted, possibly leading to biased estimates of our results. Lastly, although RI-CLPM is considered a residual-level methodology (54), which decomposes longitudinal correlations between constructs into stable person-to-person correlations and time-invariant intrapersonal dynamics (54). This decomposition allows for the estimation of within-person cross-lagged effects after adjusting for stabilizing factors (55). Many scholars argue that RI-CLPM is superior to CLPM, especially in the presence of stable factors (56–58). However, Lüdtke et al. (59), found that RI-CLPM has limited capacity to control for unobserved stable confounders when estimating cross-lagged effects. The within-person cross-lagged effect in RI-CLPM, which estimates the effect of a one-unit increase around a person’s mean, is often less relevant for testing causal hypotheses with longitudinal data, as it captures only individual temporary fluctuations and overlooks potential causes of between-person differences. Furthermore, Lüdtke’s simulation study confirms that RI-CLPM provides biased estimates of cross-lagged effects and heavily relies on specific parametric assumptions, leading to biases in different data generation scenarios (55). These critical perspectives suggest caution in interpreting our study’s results and emphasize the need to consider the inherent limitations of these models. Future research should explore improvements and alternatives to enhance the accuracy and reliability of the analysis.

## 5 Conclusion

Overall, this study supports a bidirectional relationship between SMI and SFI in colorectal cancer patients. We found that the relative strength of these relationships varies perioperatively. Specifically, SMI negatively predicted SFI from the preoperative period to 2 months postoperatively, but positively predicted SFI from 2 to 5 months postoperatively, with the effect size gradually decreasing. At 2 months postoperatively, SFI emerged as the major predictor. This suggests that age-related skeletal muscle loss increases subcutaneous adipose tissue storage, which further exacerbates skeletal muscle loss in CRC patients. Additionally, we confirmed that preoperative SFI and its combination with SMI are independent prognostic factors for CRC, validating the clinical importance of preoperative SMI and SFI in CRC patinets. Future studies should investigate SMI and SFI over long-term follow-up or in healthy populations to further elucidate these complex relationships.

## Data Availability

The original contributions presented in the study are included in the article/Supplementary material, further inquiries can be directed to the corresponding authors.
