# Supplementary material for: Bidirectional association between perioperative skeletal muscle and subcutaneous fat in colorectal cancer patients and their prognostic significance
Source: Front Nutr. 2024 Sep 18;11:1381995. doi: 10.3389/fnut.2024.1381995 (PMC11445023; doi:10.3389/fnut.2024.1381995)
Supplement: Supplementary file 1 [file Table_1.docx]

Supplementary Material

Perioperative Skeletal Muscle Mass and Subcutaneous Fat in Colorectal Cancer: A Random-Intercept Cross-Lagged Panel Study

**Guanghong Yan^1†^, Lizhu Liu^2†^, Mengmei Liu^1†^, Xinyue Jiang^3†^, Ping Chen**^1^**, Min Li**^1^**, Qingyan Ma**^1^**, Yani Li**^1^**, Sifan Duan**^1^**, Ruimin You^2^, Yanni Huang^2*^, Zhenhui Li^2*^, Dingyun You^1*^**

*** Correspondence:** Dingyun You: [youdingyun@kmmu.edu.cn](mailto:youdingyun@kmmu.edu.cn); Zhenhui Li：[lizhenhui621@qq.com](mailto:lizhenhui621@qq.com); Yanni Huang：huangyanni@kmmu.edu.cn

# Supplementary Figures and Tables

## Supplementary Figures


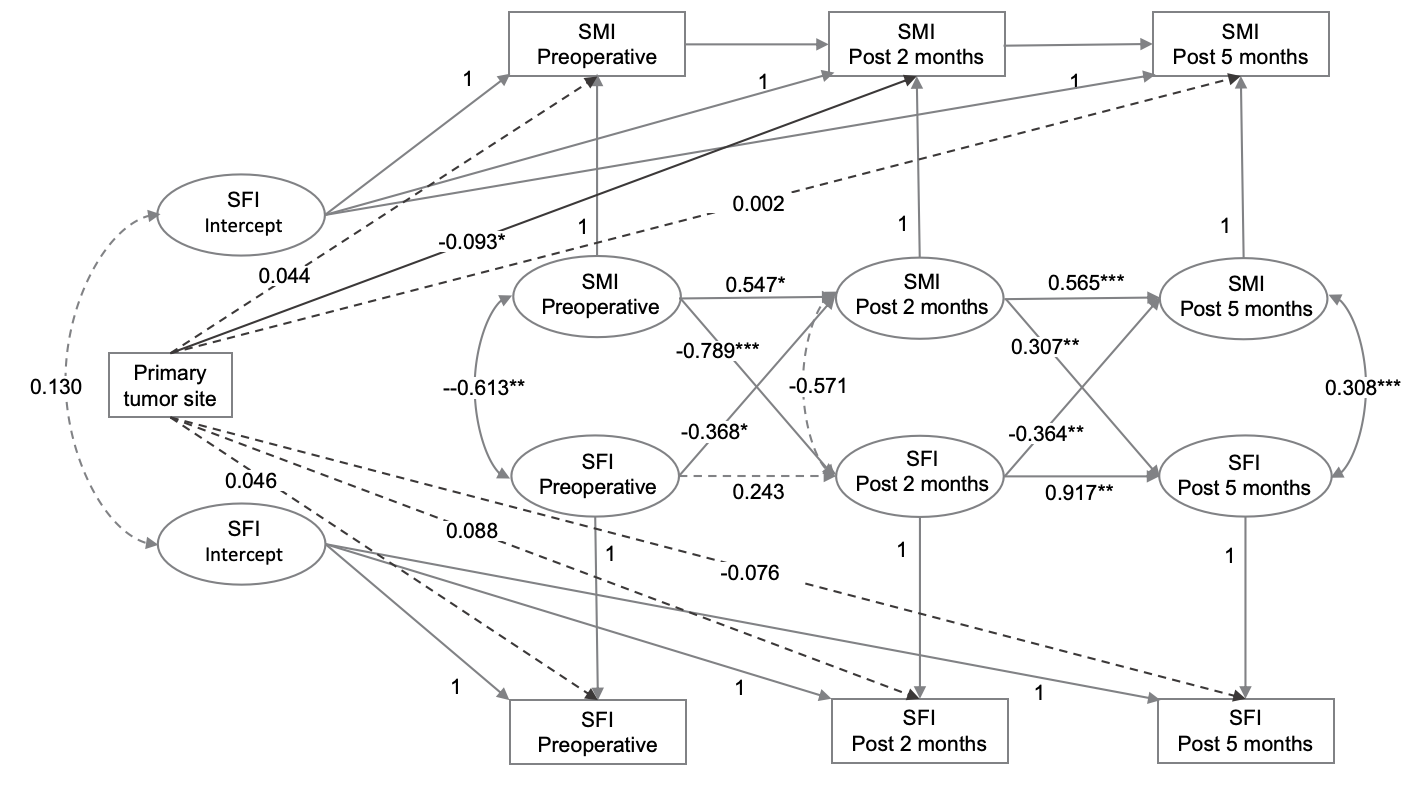


Supplementary Figure 1. Relationship between SMI and SFI analyzed preoperatively, 2 months postoperatively, and 5 months postoperatively using RI-CLPM, adjusted for preoperative primary tumor site. Solid black arrows indicate significant regression weights or correlations, while dashed arrows indicate non-significant parameters (P > 0.05). Standardised estimates are provided. Model χ²/df (1): 8.826 (P = 0.003), CFI: 0.999, TLI: 0.982, RMSEA: 0.074 [90% CI: 0.035–0.121], SRMR: 0.015.

## Supplementary Tables

Supplementary Table 1: Comparison of demographic characteristics and variables of interest in included and excluded populations.

| Variable | All Patients (n = 2580 ) | Excluded (n = 1132) | Included (n = 1448) | P value |
| --- | --- | --- | --- | --- |
| Age (Median, IQR) | 60.00 (51.00, 67.25) | 61.00 (52.00, 69.00) | 59.00 (51.00, 66.00) | <0.001 |
| Sex, (n, %) |  |  |  | 0.070 |
| Female | 1087 (42.13) | 500 (44.17) | 587 (40.54) |  |
| Male | 1493 (57.87) | 632 (55.83) | 861 (59.46) |  |
| Weight (Median, IQR) | 60.00 (54.00, 67.00) | 60.00 (53.00, 66.00) | 60.00 (54.00, 68.00) | 0.001 |
| Weight change at first postoperative follow-up (Median, IQR) | -3.00 (-5.00, -1.00) | -3.00 (-5.00, 0.00) | -3.00 (-5.00, -1.00) | 0.016 |
| BMI (Median, IQR) | 22.50 (20.76, 24.89) | 22.33 (20.62, 24.74) | 22.66 (20.76, 25.00) | 0.032 |
| Smoking history, (n, %) |  |  |  | 0.178 |
| Yes | 641 (24.84) | 261 (23.06) | 380 (26.24) |  |
| No | 1912 (74.11) | 859 (75.88) | 1053 (72.72) |  |
| Unknow | 27 (1.05) | 12 (1.06) | 15 (1.04) |  |
| Drinking history, (n, %) |  |  |  | 0.058 |
| Yes | 477 (18.49) | 195 (17.23) | 282 (19.48) |  |
| No | 2014 (78.06) | 906 (80.04) | 1108 (76.52) |  |
| Unknow | 89 (3.45) | 31 (2.74) | 58 (4.01) |  |
| Hypertension, (n, %) |  |  |  | 0.778 |
| Yes | 622 (24.11) | 279 (24.65) | 343 (23.69) |  |
| No | 1948 (75.50) | 848 (74.91) | 1100 (75.97) |  |
| Unknow | 10 (0.39) | 5 (0.44) | 5 (0.35) |  |
| Diabetes, (n, %) |  |  |  | 0.632 |
| Yes | 211 (8.18) | 86 (7.60) | 125 (8.63) |  |
| No | 2355 (91.28) | 1040 (91.87) | 1315 (90.81) |  |
| Unknow | 14 (0.54) | 6 (0.53) | 8 (0.55) |  |
| ECOG, (n, %) |  |  |  | <0.001 |
| 0 | 1338 (51.86) | 542 (47.88) | 796 (54.97) |  |
| 1 | 1121 (43.45) | 520 (45.94) | 601 (41.51) |  |
| 2 | 74 (2.87) | 52 (4.59) | 22 (1.52) |  |
| ≥3 | 47 (1.82) | 18 (1.59) | 29 (2.00) |  |
| Charlson comorbidity index, (n, %) |  |  |  | <0.001 |
| 0 | 507 (19.65) | 204 (18.02) | 303 (20.93) |  |
| 1 | 694 (26.90) | 303 (26.77) | 391 (27.00) |  |
| 2 | 762 (29.53) | 305 (26.94) | 457 (31.56) |  |
| 3 | 459 (17.79) | 233 (20.58) | 226 (15.61) |  |
| ≥4 | 147 (5.70) | 80 (7.07) | 67 (4.63) |  |
| Unknow | 11 (0.43) | 7 (0.62) | 4 (0.28) |  |
| Primary site, (n, %) |  |  |  | 0.105 |
| Colon | 1226 (47.52) | 517 (45.67) | 709 (48.96) |  |
| Rectum | 1354 (52.48) | 615 (54.33) | 739 (51.04) |  |
| Pathological stage, (n, %) |  |  |  | 0.060 |
| I | 602 (23.33) | 284 (25.09) | 318 (21.96) |  |
| II | 1026 (39.77) | 456 (40.28) | 570 (39.36) |  |
| III | 952 (36.90) | 392 (34.63) | 560 (38.67) |  |
| Tumor differentiation, (n, %) |  |  |  | <0.001 |
| Well+ Moderate | 1644 (63.72) | 668 (59.01) | 976 (67.40) |  |
| Poor | 716 (27.75) | 349 (30.83) | 367 (25.35) |  |
| Unknown | 220 (8.53) | 115 (10.16) | 105 (7.25) |  |
| Histologic type, (n, %) |  |  |  | 0.013 |
| Mucinous type | 2429 (94.15) | 1081 (95.49) | 1348 (93.09) |  |
| Non–Mucinous type | 151 (5.85) | 51 (4.51) | 100 (6.91) |  |
| T stage, (n, %) |  |  |  | 0.087 |
| T1 | 217 (8.41) | 111 (9.81) | 106 (7.32) |  |
| T2 | 477 (18.49) | 210 (18.55) | 267 (18.44) |  |
| T3 | 1760 (68.22) | 763 (67.40) | 997 (68.85) |  |
| T4 | 126 (4.88) | 48 (4.24) | 78 (5.39) |  |
| N stage, (n, %) |  |  |  | 0.119 |
| N0 | 1604 (62.17) | 723 (63.87) | 881 (60.84) |  |
| N1 | 700 (27.13) | 284 (25.09) | 416 (28.73) |  |
| N2 | 276 (10.70) | 125 (11.04) | 151 (10.43) |  |
| Lymph node yield, (n, %) |  |  |  | 0.006 |
| <12 | 543 (21.05) | 267 (23.59) | 276 (19.06) |  |
| ≥12 | 2037 (78.95) | 865 (76.41) | 1172 (80.94) |  |
| Lymph vascular invasion, (n, %) |  |  |  | 0.701 |
| Yes | 202 (7.83) | 90 (7.95) | 112 (7.73) |  |
| No | 394 (15.27) | 180 (15.90) | 214 (14.78) |  |
| Unknown | 1984 (76.90) | 862 (76.15) | 1122 (77.49) |  |
| Perineural invasion, (n, %) |  |  |  | 0.664 |
| Yes | 59 (2.29) | 27 (2.39) | 32 (2.21) |  |
| No | 503 (19.50) | 229 (20.23) | 274 (18.92) |  |
| Unknown | 2018 (78.22) | 876 (77.39) | 1142 (78.87) |  |
| Tumor deposit, (n, %) |  |  |  | <0.001 |
| Yes | 223 (8.64) | 58 (5.12) | 165 (11.40) |  |
| No | 2357 (91.36) | 1074 (94.88) | 1283 (88.60) |  |
| Adjuvant chemotherapy, (n, %) |  |  |  | <0.001 |
| Yes | 1464 (56.74) | 482 (42.58) | 982 (67.82) |  |
| No | 1115 (43.22) | 649 (57.33) | 466 (32.18) |  |
| Unknown | 1 (0.04) | 1 (0.09) | 0 (0.00) |  |
| SMI Preoperative (Median, IQR) | 43.58 (38.25, 49.58) | 42.36 (37.64, 49.01) | 44.13 (38.65, 49.76) | 0.003 |
| SMI Post 2 months (Median, IQR) | 44.08 (38.66, 49.43) | 42.37 (38.38, 46.93) | 44.15 (38.69, 49.51) | 0.262 |
| SMI Post 5 months (Median, IQR) | 45.53 (39.89, 51.53) | 43.34 (38.68, 51.75) | 45.61 (40.03, 51.51) | 0.323 |
| SFI Preoperative (Median, IQR) | 39.67 (26.29, 57.08) | 38.34 (24.65, 55.62) | 40.13 (27.95, 57.81) | 0.055 |
| SFI Post 2 months (Median, IQR) | 36.81 (25.32, 53.31) | 36.38 (22.42, 58.17) | 36.83 (25.36, 53.06) | 0.960 |
| SFI Post 5 months (Median, IQR) | 38.77 (27.53, 53.97) | 39.36 (24.99, 60.17) | 38.74 (27.63, 53.88) | 0.938 |

Note: IQR, interquartile range; BMI, Body Mass Index; ECOG, eastern cooperative oncology group; SMI, skeletal muscle index; SFI, subcutaneous fat index; Post, postoperative.

Supplementary Table 2: Comparison of demographic characteristics between those who remain in the cohort and those who are lost at 2 months postoperatively.

| Variable | All Patients (n = 2580) | Excluded (n = 1289) | Included (n = 1291) | P value |
| --- | --- | --- | --- | --- |
| Age (Median, IQR) | 60.00 (54.00, 67.00) | 60.00 (53.00, 66.00) | 60.00 (54.00, 68.00) | <0.001 |
| Sex, (n, %) |  |  |  | 0.142 |
| Female | 1087 (42.13) | 562 (43.60) | 525 (40.67) |  |
| Male | 1493 (57.87) | 727 (56.40) | 766 (59.33) |  |
| Weight (Median, IQR) | 60.00 (51.00, 67.25) | 61.00 (52.00, 69.00) | 59.00 (50.00, 66.00) | <0.001 |
| Weight change at first postoperative follow-up (Median, IQR) | -3.00 (-5.00, -1.00) | -3.00 (-5.00, 0.00) | -3.00 (-5.00, -1.00) | 0.003 |
| BMI (Median, IQR) | 22.50 (20.76, 24.89) | 22.34 (20.61, 24.61) | 22.68 (20.76, 25.12) | 0.004 |
| Smoking history, (n, %) |  |  |  | 0.033 |
| Yes | 641 (24.84) | 292 (22.65) | 349 (27.03) |  |
| No | 1912 (74.11) | 982 (76.18) | 930 (72.04) |  |
| Unknow | 27 (1.05) | 15 (1.16) | 12 (0.93) |  |
| Drinking history, (n, %) |  |  |  | 0.024 |
| Yes | 477 (18.49) | 220 (17.07) | 257 (19.91) |  |
| No | 2014 (78.06) | 1033 (80.14) | 981 (75.99) |  |
| Unknow | 89 (3.45) | 36 (2.79) | 53 (4.11) |  |
| Hypertension, (n, %) |  |  |  | 0.669 |
| Yes | 622 (24.11) | 317 (24.59) | 305 (23.63) |  |
| No | 1948 (75.50) | 966 (74.94) | 982 (76.07) |  |
| Unknow | 10 (0.39) | 6 (0.47) | 4 (0.31) |  |
| Diabetes, (n, %) |  |  |  | 0.566 |
| Yes | 211 (8.18) | 98 (7.60) | 113 (8.75) |  |
| No | 2355 (91.28) | 1184 (91.85) | 1171 (90.70) |  |
| Unknow | 14 (0.54) | 7 (0.54) | 7 (0.54) |  |
| ECOG, (n, %) |  |  |  | <0.001 |
| 0 | 1338 (51.86) | 619 (48.02) | 719 (55.69) |  |
| 1 | 1121 (43.45) | 593 (46.00) | 528 (40.90) |  |
| 2 | 74 (2.87) | 58 (4.50) | 16 (1.24) |  |
| ≥3 | 47 (1.82) | 19 (1.47) | 28 (2.17) |  |
| Charlson comorbidity index, (n, %) |  |  |  | <0.001 |
| 0 | 507 (19.65) | 227 (17.61) | 280 (21.69) |  |
| 1 | 694 (26.90) | 348 (27.00) | 346 (26.80) |  |
| 2 | 762 (29.53) | 350 (27.15) | 412 (31.91) |  |
| 3 | 459 (17.79) | 266 (20.64) | 193 (14.95) |  |
| ≥4 | 147 (5.70) | 90 (6.98) | 57 (4.42) |  |
| Unknow | 11 (0.43) | 8 (0.62) | 3 (0.23) |  |
| Primary site, (n, %) |  |  |  | 0.005 |
| Colon | 1226 (47.52) | 576 (44.69) | 650 (50.35) |  |
| Rectum | 1354 (52.48) | 713 (55.31) | 641 (49.65) |  |
| Pathological stage, (n, %) |  |  |  | <0.001 |
| I | 602 (23.33) | 345 (26.76) | 257 (19.91) |  |
| II | 1026 (39.77) | 505 (39.18) | 521 (40.36) |  |
| III | 952 (36.90) | 439 (34.06) | 513 (39.74) |  |
| Tumor differentiation, (n, %) |  |  |  | <0.001 |
| Well+ Moderate | 1644 (63.72) | 770 (59.74) | 874 (67.70) |  |
| Poor | 716 (27.75) | 388 (30.10) | 328 (25.41) |  |
| Unknown | 220 (8.53) | 131 (10.16) | 89 (6.89) |  |
| Histologic type, (n, %) |  |  |  | 0.001 |
| Mucinous type | 2429 (94.15) | 1233 (95.66) | 1196 (92.64) |  |
| Non–Mucinous type | 151 (5.85) | 56 (4.34) | 95 (7.36) |  |
| T stage, (n, %) |  |  |  | 0.001 |
| T1 | 217 (8.41) | 135 (10.47) | 82 (6.35) |  |
| T2 | 477 (18.49) | 248 (19.24) | 229 (17.74) |  |
| T3 | 1760 (68.22) | 850 (65.94) | 910 (70.49) |  |
| T4 | 126 (4.88) | 56 (4.34) | 70 (5.42) |  |
| N stage, (n, %) |  |  |  | 0.013 |
| N0 | 1604 (62.17) | 833 (64.62) | 771 (59.72) |  |
| N1 | 700 (27.13) | 317 (24.59) | 383 (29.67) |  |
| N2 | 276 (10.70) | 139 (10.78) | 137 (10.61) |  |
| Lymph node yield, (n, %) |  |  |  | 0.019 |
| <12 | 543 (21.05) | 296 (22.96) | 247 (19.13) |  |
| ≥12 | 2037 (78.95) | 993 (77.04) | 1044 (80.87) |  |
| Lymph vascular invasion, (n, %) |  |  |  | 0.938 |
| Yes | 202 (7.83) | 100 (7.76) | 102 (7.90) |  |
| No | 394 (15.27) | 200 (15.52) | 194 (15.03) |  |
| Unknown | 1984 (76.90) | 989 (76.73) | 995 (77.07) |  |
| Perineural invasion, (n, %) |  |  |  | 0.959 |
| Yes | 59 (2.29) | 29 (2.25) | 30 (2.32) |  |
| No | 503 (19.50) | 254 (19.71) | 249 (19.29) |  |
| Unknown | 2018 (78.22) | 1006 (78.04) | 1012 (78.39) |  |
| Tumor deposit, (n, %) |  |  |  | <0.001 |
| Yes | 223 (8.64) | 69 (5.35) | 154 (11.93) |  |
| No | 2357 (91.36) | 1220 (94.65) | 1137 (88.07) |  |
| Adjuvant chemotherapy, (n, %) |  |  |  | <0.001 |
| Yes | 1464 (56.74) | 546 (42.36) | 918 (71.11) |  |
| No | 1115 (43.22) | 742 (57.56) | 373 (28.89) |  |
| Unknown | 1 (0.04) | 1 (0.08) | 0 (0.00) |  |
| SMI Preoperative (Median, IQR) | 43.58 (38.25, 49.58) | 42.53 (37.65, 48.93) | 44.19 (38.73, 49.94) | 0.001 |
| SMI Post 2 months (Median, IQR) | 44.08 (38.66, 49.43) | 42.37 (38.38, 46.93) | 44.15 (38.69, 49.51) | 0.262 |
| SMI Post 5 months (Median, IQR) | 45.53 (39.89, 51.53) | 44.68 (39.04, 50.26) | 45.74 (40.00, 51.70) | 0.170 |
| SFI Preoperative (Median, IQR) | 39.67 (26.29, 57.08) | 38.54 (24.88, 55.54) | 40.21 (28.30, 58.58) | 0.029 |
| SFI Post 2 months (Median, IQR) | 36.81 (25.32, 53.31) | 36.38 (22.42, 58.17) | 36.83 (25.36, 53.06) | 0.960 |
| SFI Post 5 months (Median, IQR) | 38.77 (27.53, 53.97) | 38.66 (27.83, 54.59) | 38.78 (27.53, 53.81) | 0.724 |

Note: IQR, interquartile range; BMI, Body Mass Index; ECOG, eastern cooperative oncology group; SMI, skeletal muscle index; SFI, subcutaneous fat index; Post, postoperative.

Supplementary Table 3: Comparison of demographic characteristics between those who remain in the cohort and those who are lost at 5 months postoperatively.

| Variable | All Patients (n = 2580 ) | Excluded (n = 1647) | Included (n = 933) | P value |
| --- | --- | --- | --- | --- |
| Age (Median, IQR) | 60.00 (51.00, 67.25) | 61.00 (52.00, 68.00) | 59.00 (50.00, 65.00) | <0.001 |
| Sex, (n, %) |  |  |  | 0.034 |
| Female | 1087 (42.13) | 720 (43.72) | 367 (39.34) |  |
| Male | 1493 (57.87) | 927 (56.28) | 566 (60.66) |  |
| Weight (Median, IQR) | 60.00 (54.00, 67.00) | 60.00 (53.00, 66.00) | 60.00 (55.00, 68.00) | <0.001 |
| Weight change at first postoperative follow-up (Median, IQR) | -3.00 (-5.00, -1.00) | -3.00 (-5.00, 0.00) | -3.00 (-5.00, -1.00) | 0.055 |
| BMI (Median, IQR) | 22.50 (20.76, 24.89) | 22.43 (20.70, 24.74) | 22.66 (20.76, 25.10) | 0.044 |
| Smoking history, (n, %) |  |  |  | 0.236 |
| Yes | 641 (24.84) | 395 (23.98) | 246 (26.37) |  |
| No | 1912 (74.11) | 1232 (74.80) | 680 (72.88) |  |
| Unknow | 27 (1.05) | 20 (1.21) | 7 (0.75) |  |
| Drinking history, (n, %) |  |  |  | 0.414 |
| Yes | 477 (18.49) | 292 (17.73) | 185 (19.83) |  |
| No | 2014 (78.06) | 1297 (78.75) | 717 (76.85) |  |
| Unknow | 89 (3.45) | 58 (3.52) | 31 (3.32) |  |
| Hypertension, (n, %) |  |  |  | 0.461 |
| Yes | 622 (24.11) | 404 (24.53) | 218 (23.37) |  |
| No | 1948 (75.50) | 1235 (74.98) | 713 (76.42) |  |
| Unknow | 10 (0.39) | 8 (0.49) | 2 (0.21) |  |
| Diabetes, (n, %) |  |  |  | 0.504 |
| Yes | 211 (8.18) | 136 (8.26) | 75 (8.04) |  |
| No | 2355 (91.28) | 1500 (91.07) | 855 (91.64) |  |
| Unknow | 14 (0.54) | 11 (0.67) | 3 (0.32) |  |
| ECOG, (n, %) |  |  |  | 0.008 |
| 0 | 1338 (51.86) | 830 (50.39) | 508 (54.45) |  |
| 1 | 1121 (43.45) | 727 (44.14) | 394 (42.23) |  |
| 2 | 74 (2.87) | 60 (3.64) | 14 (1.50) |  |
| ≥3 | 47 (1.82) | 30 (1.82) | 17 (1.82) |  |
| Charlson comorbidity index, (n, %) |  |  |  | <0.001 |
| 0 | 507 (19.65) | 300 (18.21) | 207 (22.19) |  |
| 1 | 694 (26.90) | 432 (26.23) | 262 (28.08) |  |
| 2 | 762 (29.53) | 468 (28.42) | 294 (31.51) |  |
| 3 | 459 (17.79) | 326 (19.79) | 133 (14.26) |  |
| ≥4 | 147 (5.70) | 111 (6.74) | 36 (3.86) |  |
| Unknow | 11 (0.43) | 10 (0.61) | 1 (0.11) |  |
| Primary site, (n, %) |  |  |  | 0.185 |
| Colon | 1226 (47.52) | 766 (46.51) | 460 (49.30) |  |
| Rectum | 1354 (52.48) | 881 (53.49) | 473 (50.70) |  |
| Pathological stage, (n, %) |  |  |  | <0.001 |
| I | 602 (23.33) | 435 (26.41) | 167 (17.90) |  |
| II | 1026 (39.77) | 642 (38.98) | 384 (41.16) |  |
| III | 952 (36.90) | 570 (34.61) | 382 (40.94) |  |
| Tumor differentiation, (n, %) |  |  |  | 0.006 |
| Well+ Moderate | 1644 (63.72) | 1021 (61.99) | 623 (66.77) |  |
| Poor | 716 (27.75) | 466 (28.29) | 250 (26.80) |  |
| Unknown | 220 (8.53) | 160 (9.71) | 60 (6.43) |  |
| Histologic type, (n, %) |  |  |  | 0.003 |
| Mucinous type | 2429 (94.15) | 1568 (95.20) | 861 (92.28) |  |
| Non–Mucinous type | 151 (5.85) | 79 (4.80) | 72 (7.72) |  |
| T stage, (n, %) |  |  |  | <0.001 |
| T1 | 217 (8.41) | 167 (10.14) | 50 (5.36) |  |
| T2 | 477 (18.49) | 324 (19.67) | 153 (16.40) |  |
| T3 | 1760 (68.22) | 1082 (65.70) | 678 (72.67) |  |
| T4 | 126 (4.88) | 74 (4.49) | 52 (5.57) |  |
| N stage, (n, %) |  |  |  | 0.015 |
| N0 | 1604 (62.17) | 1057 (64.18) | 547 (58.63) |  |
| N1 | 700 (27.13) | 418 (25.38) | 282 (30.23) |  |
| N2 | 276 (10.70) | 172 (10.44) | 104 (11.15) |  |
| Lymph node yield, (n, %) |  |  |  | 0.003 |
| <12 | 543 (21.05) | 377 (22.89) | 166 (17.79) |  |
| ≥12 | 2037 (78.95) | 1270 (77.11) | 767 (82.21) |  |
| Lymph vascular invasion, (n, %) |  |  |  | 0.118 |
| Yes | 202 (7.83) | 117 (7.10) | 85 (9.11) |  |
| No | 394 (15.27) | 262 (15.91) | 132 (14.15) |  |
| Unknown | 1984 (76.90) | 1268 (76.99) | 716 (76.74) |  |
| Perineural invasion, (n, %) |  |  |  | 0.658 |
| Yes | 59 (2.29) | 41 (2.49) | 18 (1.93) |  |
| No | 503 (19.50) | 320 (19.43) | 183 (19.61) |  |
| Unknown | 2018 (78.22) | 1286 (78.08) | 732 (78.46) |  |
| Tumor deposit, (n, %) |  |  |  | <0.001 |
| Yes | 223 (8.64) | 105 (6.38) | 118 (12.65) |  |
| No | 2357 (91.36) | 1542 (93.62) | 815 (87.35) |  |
| Adjuvant chemotherapy, (n, %) |  |  |  | <0.001 |
| Yes | 1464 (56.74) | 770 (46.75) | 694 (74.38) |  |
| No | 1115 (43.22) | 876 (53.19) | 239 (25.62) |  |
| Unknown | 1 (0.04) | 1 (0.06) | 0 (0.00) |  |
| SMI Preoperative (Median, IQR) | 43.58 (38.25, 49.58) | 43.01 (37.99, 49.30) | 44.43 (38.65, 50.03) | 0.007 |
| SMI Post 2 months (Median, IQR) | 44.08 (38.66, 49.43) | 43.71 (38.51, 48.88) | 44.47 (38.83, 49.86) | 0.079 |
| SMI Post 5 months (Median, IQR) | 45.53 (39.89, 51.53) | 43.34 (38.68, 51.75) | 45.61 (40.03, 51.51) | 0.323 |
| SFI Preoperative (Median, IQR) | 39.67 (26.29, 57.08) | 39.11 (24.77, 57.07) | 40.16 (28.62, 57.44) | 0.117 |
| SFI Post 2 months (Median, IQR) | 36.81 (25.32, 53.31) | 36.92 (25.00, 55.67) | 36.75 (25.49, 51.83) | 0.858 |
| SFI Post 5 months (Median, IQR) | 38.77 (27.53, 53.97) | 39.36 (24.99, 60.17) | 38.74 (27.63, 53.88) | 0.938 |

Note: IQR, interquartile range; BMI, Body Mass Index; ECOG, eastern cooperative oncology group; SMI, skeletal muscle index; SFI, subcutaneous fat index; Post, postoperative.

Supplementary Table 4: Demographic and clinical characteristics stratified by overall survival status and recurrence-free survival status.

| Variable | All (n = 1448) | Overall Survival Status | | |  | Recurrence-Free Survival Status | | |
| --- | --- | --- | --- | --- | --- | --- | --- | --- |
|  |  | Survivors (n = 1263) | Non-survivors (n = 185) | P value |  | No Recurrence (n = 1073) | Recurrence (n = 375) | P value |
| Age (Median, IQR) | 59.00 (51.00,66.00) | 59.00 (51.00,66.00) | 61.00 (50.00,68.00) | 0.065 |  | 59.00 (51.00,66.00) | 60.00 (50.00,66.00) | 0.984 |
| Sex, (n, %) |  |  |  | 0.810 |  |  |  | 0.953 |
| Female | 587 (40.54) | 514 (40.70) | 73 (39.46) |  |  | 434 (40.45) | 153 (40.80) |  |
| Male | 861 (59.46) | 749 (59.30) | 112 (60.54) |  |  | 639 (59.55) | 222 (59.20) |  |
| Weight (Median, IQR) | 61.09 (10.30) | 61.18 (10.29) | 60.51 (10.38) | 0.416 |  | 61.08 (10.40) | 61.12 (10.02) | 0.949 |
| Weight change at first postoperative follow-up (Median, IQR) | -3.00 (-5.00,-1.00) | -3.00 (-5.00,-1.00) | -3.00 (-5.00,0.00) | 0.064 |  | -3.00 (-5.00,-1.00) | -3.00 (-5.00,-0.50) | 0.429 |
| BMI (Median, IQR) | 22.66 (20.76,25.00) | 22.66 (20.76,24.99) | 22.72 (20.76,25.16) | 0.807 |  | 22.59 (20.76,24.86) | 22.86 (20.76,25.39) | 0.478 |
| Smoking history, (n, %) |  |  |  | 0.512 |  |  |  | 0.290 |
| Yes | 380 (26.24) | 335 (26.52) | 45 (24.32) |  |  | 292 (27.21) | 88 (23.47) |  |
| No | 1053 (72.72) | 916 (72.53) | 137 (74.05) |  |  | 771 (71.85) | 282 (75.20) |  |
| Unknow | 15 (1.04) | 12 (0.95) | 3 (1.62) |  |  | 10 (0.93) | 5 (1.33) |  |
| Drinking history, (n, %) |  |  |  | 0.704 |  |  |  | 0.953 |
| Yes | 282 (19.48) | 250 (19.79) | 32 (17.30) |  |  | 211 (19.66) | 71 (18.93) |  |
| No | 1108 (76.52) | 962 (76.17) | 146 (78.92) |  |  | 819 (76.33) | 289 (77.07) |  |
| Unknow | 58 (4.01) | 51 (4.04) | 7 (3.78) |  |  | 43 (4.01) | 15 (4.00) |  |
| Hypertension, (n, %) |  |  |  | 0.963 |  |  |  | 0.484 |
| Yes | 343 (23.69) | 300 (23.75) | 43 (23.24) |  |  | 257 (23.95) | 86 (22.93) |  |
| No | 1100 (75.97) | 958 (75.85) | 142 (76.76) |  |  | 811 (75.58) | 289 (77.07) |  |
| Unknow | 5 (0.35) | 5 (0.40) | 0 (0.00) |  |  | 5 (0.47) | 0 (0.00) |  |
| Diabetes, (n, %) |  |  |  | 0.562 |  |  |  | 0.851 |
| Yes | 125 (8.63) | 112 (8.87) | 13 (7.03) |  |  | 93 (8.67) | 32 (8.53) |  |
| No | 1315 (90.81) | 1143 (90.50) | 172 (92.97) |  |  | 973 (90.68) | 342 (91.20) |  |
| Unknow | 8 (0.55) | 8 (0.63) | 0 (0.00) |  |  | 7 (0.65) | 1 (0.27) |  |
| ECOG, (n, %) |  |  |  | 0.002 |  |  |  | 0.008 |
| 0 | 796 (54.97) | 705 (55.82) | 91 (49.19) |  |  | 610 (56.85) | 186 (49.60) |  |
| 1 | 601 (41.51) | 522 (41.33) | 79 (42.70) |  |  | 433 (40.35) | 168 (44.80) |  |
| 2 | 22 (1.52) | 18 (1.43) | 4 (2.16) |  |  | 15 (1.40) | 7 (1.87) |  |
| ≥3 | 29 (2.00) | 18 (1.43) | 11 (5.95) |  |  | 15 (1.40) | 14 (3.73) |  |
| Charlson comorbidity index, (n, %) |  |  |  | 0.100 |  |  |  | 0.714 |
| 0 | 303 (20.93) | 260 (20.59) | 43 (23.24) |  |  | 218 (20.32) | 85 (22.67) |  |
| 1 | 391 (27.00) | 355 (28.11) | 36 (19.46) |  |  | 298 (27.77) | 93 (24.80) |  |
| 2 | 457 (31.56) | 396 (31.35) | 61 (32.97) |  |  | 338 (31.50) | 119 (31.73) |  |
| 3 | 226 (15.61) | 188 (14.89) | 38 (20.54) |  |  | 164 (15.28) | 62 (16.53) |  |
| ≥4 | 67 (4.63) | 60 (4.75) | 7 (3.78) |  |  | 51 (4.75) | 16 (4.27) |  |
| Unknow | 4 (0.28) | 4 (0.32) | 0 (0.00) |  |  | 4 (0.37) | 0 (0.00) |  |
| Primary site, (n, %) |  |  |  | 0.423 |  |  |  | 0.274 |
| Colon | 709 (48.96) | 624 (49.41) | 85 (45.95) |  |  | 535 (49.86) | 174 (46.40) |  |
| Rectum | 739 (51.04) | 639 (50.59) | 100 (54.05) |  |  | 538 (50.14) | 201 (53.60) |  |
| Pathological stage, (n, %) |  |  |  | <0.001 |  |  |  | <0.001 |
| I | 318 (21.96) | 299 (23.67) | 19 (10.27) |  |  | 268 (24.98) | 50 (13.33) |  |
| II | 570 (39.36) | 530 (41.96) | 40 (21.62) |  |  | 471 (43.90) | 99 (26.40) |  |
| III | 560 (38.67) | 434 (34.36) | 126 (68.11) |  |  | 334 (31.13) | 226 (60.27) |  |
| Tumor differentiation, (n, %) |  |  |  | 0.007 |  |  |  | 0.024 |
| Well+ Moderate | 976 (67.40) | 865 (68.49) | 111 (60.00) |  |  | 737 (68.69) | 239 (63.73) |  |
| Poor | 367 (25.35) | 303 (23.99) | 64 (34.59) |  |  | 253 (23.58) | 114 (30.40) |  |
| Unknown | 105 (7.25) | 95 (7.52) | 10 (5.41) |  |  | 83 (7.74) | 22 (5.87) |  |
| Histologic type, (n, %) |  |  |  | 0.932 |  |  |  | 0.887 |
| Mucinous type | 1348 (93.09) | 1175 (93.03) | 173 (93.51) |  |  | 1000 (93.20) | 348 (92.80) |  |
| Non–Mucinous type | 100 (6.91) | 88 (6.97) | 12 (6.49) |  |  | 73 (6.80) | 27 (7.20) |  |
| T stage, (n, %) |  |  |  | <0.001 |  |  |  | <0.001 |
| T1 | 106 (7.32) | 103 (8.16) | 3 (1.62) |  |  | 95 (8.85) | 11 (2.93) |  |
| T2 | 267 (18.44) | 244 (19.32) | 23 (12.43) |  |  | 215 (20.04) | 52 (13.87) |  |
| T3 | 997 (68.85) | 846 (66.98) | 151 (81.62) |  |  | 703 (65.52) | 294 (78.40) |  |
| T4 | 78 (5.39) | 70 (5.54) | 8 (4.32) |  |  | 60 (5.59) | 18 (4.80) |  |
| N stage, (n, %) |  |  |  | <0.001 |  |  |  | <0.001 |
| N0 | 881 (60.84) | 822 (65.08) | 59 (31.89) |  |  | 733 (68.31) | 148 (39.47) |  |
| N1 | 416 (28.73) | 337 (26.68) | 79 (42.70) |  |  | 271 (25.26) | 145 (38.67) |  |
| N2 | 151 (10.43) | 104 (8.23) | 47 (25.41) |  |  | 69 (6.43) | 82 (21.87) |  |
| Lymph node yield, (n, %) |  |  |  | 0.724 |  |  |  | 0.997 |
| <12 | 276 (19.06) | 243 (19.24) | 33 (17.84) |  |  | 204 (19.01) | 72 (19.20) |  |
| ≥12 | 1172 (80.94) | 1020 (80.76) | 152 (82.16) |  |  | 869 (80.99) | 303 (80.80) |  |
| Lymph vascular invasion, (n, %) |  |  |  | <0.001 |  |  |  | <0.001 |
| Yes | 112 (7.73) | 78 (6.18) | 34 (18.38) |  |  | 56 (5.22) | 56 (14.93) |  |
| No | 214 (14.78) | 195 (15.44) | 19 (10.27) |  |  | 172 (16.03) | 42 (11.20) |  |
| Unknown | 1122 (77.49) | 990 (78.38) | 132 (71.35) |  |  | 845 (78.75) | 277 (73.87) |  |
| Perineural invasion, (n, %) |  |  |  | <0.001 |  |  |  | <0.001 |
| Yes | 32 (2.21) | 18 (1.43) | 14 (7.57) |  |  | 11 (1.03) | 21 (5.60) |  |
| No | 274 (18.92) | 240 (19.00) | 34 (18.38) |  |  | 205 (19.11) | 69 (18.40) |  |
| Unknown | 1142 (78.87) | 1005 (79.57) | 137 (74.05) |  |  | 857 (79.87) | 285 (76.00) |  |
| Tumor deposit, (n, %) |  |  |  | <0.001 |  |  |  | <0.001 |
| Yes | 165 (11.40) | 117 (9.26) | 48 (25.95) |  |  | 81 (7.55) | 84 (22.40) |  |
| No | 1283 (88.60) | 1146 (90.74) | 137 (74.05) |  |  | 992 (92.45) | 291 (77.60) |  |
| Adjuvant chemotherapy, (n, %) |  |  |  | 0.007 |  |  |  | <0.001 |
| Yes | 982 (67.82) | 840 (66.51) | 142 (76.76) |  |  | 692 (64.49) | 290 (77.33) |  |
| No | 466 (32.18) | 423 (33.49) | 43 (23.24) |  |  | 381 (35.51) | 85 (22.67) |  |
| SMI Preoperative (Median, IQR) | 44.13 (38.65,49.76) | 44.20 (38.62,49.87) | 43.71 (39.01,49.18) | 0.767 |  | 44.08 (38.69,49.69) | 44.22 (38.52,50.19) | 0.871 |
| SMI Post 2 months (Median, IQR) | 44.15 (38.69,49.51) | 44.15 (38.65,49.59) | 44.12 (39.16,49.35) | 0.781 |  | 44.19 (38.74,49.67) | 44.07 (38.45,48.95) | 0.377 |
| SMI Post 5 months (Median, IQR) | 45.61 (40.03,51.51) | 45.53 (40.06,51.39) | 45.83 (39.37,52.42) | 0.686 |  | 45.47 (40.22,51.42) | 45.82 (39.10,51.77) | 0.881 |
| SFI Preoperative (Median, IQR) | 40.13 (27.95,57.81) | 40.22 (28.34,57.79) | 37.96 (24.65,58.75) | 0.373 |  | 40.01 (27.66,57.61) | 40.74 (28.39,58.67) | 0.672 |
| SFI Post 2 months (Median, IQR) | 36.83 (25.36,53.06) | 36.70 (25.49,52.58) | 39.80 (23.15,56.34) | 0.950 |  | 36.22 (25.41,52.56) | 39.26 (25.30,53.95) | 0.406 |
| SFI Post 5 months (Median, IQR) | 38.74 (27.63,53.88) | 38.83 (27.91,53.64) | 35.40 (26.89,54.82) | 0.343 |  | 38.87 (27.96,53.58) | 38.16 (27.11,54.90) | 0.533 |

Note: IQR, interquartile range; BMI, Body Mass Index; ECOG, eastern cooperative oncology group; SMI, skeletal muscle index; SFI, subcutaneous fat index; Post, postoperative

Supplementary Table 5: Univariate analysis of clinical variables, preoperative baseline, and postoperative skeletal muscle and subcutaneous fat indices at 2 and 5 months in relation to recurrence-free survival**.**

| Variable | HR (95%CI) | P value |
| --- | --- | --- |
| Age | 1.00 (0.99, 1.01) | 0.609 |
| Sex |  |  |
| Female | Ref |  |
| Male | 0.97 (0.79, 1.19) | 0.777 |
| BMI | 1.01 (0.98, 1.04) | 0.583 |
| Smoking history |  |  |
| Yes | Ref |  |
| No | 1.18 (0.93, 1.50) | 0.180 |
| Unknow | 1.50 (0.61, 3.70) | 0.375 |
| Drinking history |  |  |
| Yes | Ref |  |
| No | 1.03 (0.80, 1.34) | 0.797 |
| Unknow | 1.07 (0.61, 1.87) | 0.809 |
| Hypertension |  |  |
| Yes | Ref |  |
| No | 1.04 (0.81, 1.32) | 0.769 |
| Diabetes |  |  |
| Yes | Ref |  |
| No | 1.00 (0.69, 1.43) | 0.980 |
| ECOG |  |  |
| 0 | Ref |  |
| 1 | 1.22 (0.99, 1.51) | 0.057 |
| 2 | 1.31 (0.61, 2.78) | 0.485 |
| ≥3 | 2.14 (1.24, 3.70) | 0.006 |
| Charlson comorbidity index |  |  |
| 0 | Ref |  |
| 1 | 0.82 (0.61, 1.10) | 0.185 |
| 2 | 0.93 (0.70, 1.23) | 0.598 |
| 3 | 0.98 (0.71, 1.36) | 0.904 |
| ≥4 | 0.83 (0.49, 1.42) | 0.493 |
| Primary site |  |  |
| Colon | Ref |  |
| Rectum | 1.11 (0.91, 1.36) | 0.318 |
| Pathological stage |  |  |
| I | Ref |  |
| II | 1.14 (0.81, 1.60) | 0.449 |
| III | 3.13 (2.31, 4.26) | < 0.001 |
| Tumor differentiation |  |  |
| Well+ Moderate | Ref |  |
| Poor | 1.28 (1.03, 1.60) | 0.028 |
| Unknown | 0.85 (0.55, 1.32) | 0.480 |
| Histologic type |  |  |
| Mucinous type | Ref |  |
| Non–Mucinous type | 1.16 (0.78, 1.72) | 0.459 |
| Lymph node yield |  |  |
| <12 | Ref |  |
| ≥12 | 1.02 (0.79, 1.31) | 0.904 |
| Lymph vascular invasion |  |  |
| Yes | Ref |  |
| No | 0.30 (0.20, 0.44) | < 0.001 |
| Unknown | 0.40 (0.30, 0.53) | < 0.001 |
| Perineural invasion |  |  |
| Yes | Ref |  |
| No | 0.26 (0.16, 0.42) | < 0.001 |
| Unknown | 0.26 (0.17, 0.40) | < 0.001 |
| Tumor deposit |  |  |
| Yes | Ref |  |
| No | 0.34 (0.27, 0.44) | < 0.001 |
| Adjuvant chemotherapy |  |  |
| Yes | Ref |  |
| No | 0.61 (0.48, 0.77) | < 0.001 |
| SMI Preoperative |  |  |
| Normal group | Ref |  |
| Low group | 1.05 (0.83, 1.32) | 0.689 |
| SMI Post 2 months |  |  |
| Normal group | Ref |  |
| Low group | 1.06 (0.83, 1.36) | 0.633 |
| SMI Post 5 months |  |  |
| Normal group | Ref |  |
| Low group | 1.01 (0.74, 1.39) | 0.929 |
| SFI Preoperative |  |  |
| Normal group | Ref |  |
| Low group | 1.28 (1.03, 1.59) | 0.027 |
| SFI Post 2 months |  |  |
| Normal group | Ref |  |
| Low group | 1.18 (0.92, 1.52) | 0.195 |
| SFI Post 5 months |  |  |
| Normal group | Ref |  |
| Low group | 1.16 (0.87, 1.54) | 0.323 |
| Preoperative Normal SMI and Normal SFI |  |  |
| Both | Ref |  |
| Neither | 1.25 (1.02, 1.54) | 0.028 |
| Postoperative 2 months Normal SMI and Normal SFI |  |  |
| Both | Ref |  |
| Neither | 1.18 (0.95, 1.46) | 0.136 |
| Postoperative 5 months Normal SMI and Normal SFI |  |  |
| Both | Ref |  |
| Neither | 1.12 (0.86, 1.44) | 0.401 |

Note: CI, confidence interval; HR, hazard ratio; Ref, reference; BMI, Body Mass Index; ECOG, eastern cooperative oncology group; SMI, skeletal muscle index; SFI, subcutaneous fat index.

Supplementary Table 6: Univariate analysis of clinical variables, preoperative baseline, and postoperative skeletal muscle and subcutaneous fat indices at 2 and 5 months in relation to overall survival**.**

| Variable | HR (95%CI) | P value |
| --- | --- | --- |
| Age | 1.01 (1.00, 1.02) | 0.117 |
| Sex |  |  |
| Female | Ref |  |
| Male | 1.04 (0.77, 1.40) | 0.795 |
| BMI | 0.99 (0.95, 1.04) | 0.768 |
| Smoking history |  |  |
| Yes | Ref |  |
| No | 1.04 (0.74, 1.45) | 0.841 |
| Unknow | 1.73 (0.54, 5.58) | 0.357 |
| Drinking history |  |  |
| Yes | Ref |  |
| No | 1.10 (0.75, 1.61) | 0.624 |
| Unknow | 1.08 (0.48, 2.45) | 0.852 |
| Hypertension |  |  |
| Yes | Ref |  |
| No | 1.02 (0.73, 1.44) | 0.894 |
| Diabetes |  |  |
| Yes | Ref |  |
| No | 1.25 (0.71, 2.19) | 0.440 |
| ECOG |  |  |
| 0 | Ref |  |
| 1 | 1.16 (0.86, 1.57) | 0.334 |
| 2 | 1.40 (0.51, 3.83) | 0.507 |
| ≥3 | 3.03 (1.61, 5.70) | < 0.001 |
| Charlson comorbidity index |  |  |
| 0 | Ref |  |
| 1 | 0.66 (0.42, 1.02) | 0.062 |
| 2 | 0.98 (0.66, 1.44) | 0.901 |
| 3 | 1.21 (0.78, 1.88) | 0.383 |
| ≥4 | 0.74 (0.33, 1.65) | 0.461 |
| Primary site |  |  |
| Colon | Ref |  |
| Rectum | 1.13 (0.85, 1.51) | 0.402 |
| Pathological stage |  |  |
| I | Ref |  |
| II | 1.19 (0.69, 2.05) | 0.534 |
| III | 4.21 (2.60, 6.81) | < 0.001 |
| Tumor differentiation |  |  |
| Well+ Moderate | Ref |  |
| Poor | 1.50 (1.10, 2.04) | 0.010 |
| Unknown | 0.84 (0.44, 1.61) | 0.607 |
| Histologic type |  |  |
| Mucinous type | Ref |  |
| Non–Mucinous type | 1.16 (0.65, 2.09) | 0.620 |
| Lymph node yield |  |  |
| <12 | Ref |  |
| ≥12 | 1.14 (0.78, 1.65) | 0.510 |
| Lymph vascular invasion |  |  |
| Yes | Ref |  |
| No | 0.23 (0.13, 0.40) | < 0.001 |
| Unknown | 0.34 (0.24, 0.50) | < 0.001 |
| Perineural invasion |  |  |
| Yes | Ref |  |
| No | 0.20 (0.11, 0.38) | < 0.001 |
| Unknown | 0.21 (0.12, 0.37) | < 0.001 |
| Tumor deposit |  |  |
| Yes | Ref |  |
| No | 0.30 (0.22, 0.42) | < 0.001 |
| Adjuvant chemotherapy |  |  |
| Yes | Ref |  |
| No | 0.69 (0.49, 0.97) | 0.031 |
| SMI Preoperative |  |  |
| Normal group | Ref |  |
| Low group | 1.31 (0.96, 1.80) | 0.088 |
| SMI Post 2 months |  |  |
| Normal group | Ref |  |
| Low group | 1.11 (0.77, 1.59) | 0.571 |
| SMI Post 5 months |  |  |
| Normal group | Ref |  |
| Low group | 1.10 (0.70, 1.74) | 0.673 |
| SFI Preoperative |  |  |
| Normal group | Ref |  |
| Low group | 1.28 (0.94, 1.74) | 0.121 |
| SFI Post 2 months |  |  |
| Normal group | Ref |  |
| Low group | 1.17 (0.81, 1.70) | 0.400 |
| SFI Post 5 months |  |  |
| Normal group | Ref |  |
| Low group | 1.03 (0.67, 1.59) | 0.889 |
| Preoperative Normal SMI and Normal SFI |  |  |
| Both | Ref |  |
| Neither | 1.42 (1.06, 1.90) | 0.017 |
| Postoperative 2 months Normal SMI and Normal SFI |  |  |
| Both | Ref |  |
| Neither | 1.21 (0.88, 1.66) | 0.231 |
| Postoperative 5 months Normal SMI and Normal SFI |  |  |
| Both | Ref |  |
| Neither | 1.07 (0.74, 1.56) | 0.704 |

Note: CI, confidence interval; HR, hazard ratio; Ref, reference; BMI, Body Mass Index; ECOG, eastern cooperative oncology group; SMI, skeletal muscle index; SFI, subcutaneous fat index.

Supplementary Table 7: **Univariate and multivariate analysis of skeletal muscle index and subcutaneous fat index in relation to recurrence free survival at preoperative baseline, postoperative 2, 5 months.**

| Variable | Univariate Cox model | |  | Multivariate Cox model | |
| --- | --- | --- | --- | --- | --- |
|  | Crude HR (95% CI) | P value |  | Adjusted HR (95% CI) | P value |
| Preoperative baseline |  |  |  |  |  |
| SMI |  |  |  |  |  |
| Low group | Ref |  |  | Ref |  |
| Normal group | 1.05 (0.83, 1.32) | 0.689 |  | 1.09 (0.86, 1.38) | 0.500 |
| SFI |  |  |  |  |  |
| Normal group | Ref |  |  | Ref |  |
| High group | 1.28 (1.03, 1.59) | 0.027 |  | 1.24 (1.00, 1.55) | 0.050 |
| SMI and SFI |  |  |  |  |  |
| Both | Ref |  |  | Ref |  |
| Neither | 1.25 (1.02, 1.54) | 0.029 |  | 1.26 (1.03, 1.55) | 0.026 |
| Postoperative 2 months |  |  |  |  |  |
| SMI |  |  |  |  |  |
| Low group | Ref |  |  | Ref |  |
| Normal group | 1.06 (0.83, 1.36) | 0.633 |  | 1.07 (0.83, 1.37) | 0.605 |
| SFI |  |  |  |  |  |
| Normal group | Ref |  |  | Ref |  |
| High group | 1.18 (0.92,1.52) | 0.195 |  | 1.18 (0.92,1.53) | 0.196 |
| SMI and SFI |  |  |  |  |  |
| Both | Ref |  |  | Ref |  |
| Neither | 1.18 (0.95, 1.46) | 0.136 |  | 1.18 (0.95, 1.46) | 0.141 |
| Postoperative 5 months |  |  |  |  |  |
| SMI |  |  |  |  |  |
| Low group | Ref |  |  | Ref |  |
| Normal group | 1.01 (0.74, 1.39) | 0.929 |  | 1.05 (0.76, 1.45) | 0.768 |
| SFI |  |  |  |  |  |
| Normal group | Ref |  |  | Ref |  |
| High group | 1.16 (0.87, 1.54) | 0.323 |  | 1.09 (0.81, 1.46) | 0.567 |
| Normal SMI and Normal SFI |  |  |  |  |  |
| Both | Ref |  |  | Ref |  |
| Neither | 1.12 (0.86, 1.44) | 0.401 |  | 1.09 (0.85, 1.41) | 0.492 |

Note: CI, confidence interval; HR, hazard ratio; Ref, reference; BMI, Body Mass Index; ECOG, eastern cooperative oncology group; SMI, skeletal muscle index; SFI, subcutaneous fat index;

Multivariate Cox model was adjusted for ECOG, pathological stage, tumor differentiation, lymph vascular invasion, perineural invasion, tumor deposit and adjuvant chemotherapy.

Supplementary Table 8: **Univariate and multivariate analysis of skeletal muscle index and subcutaneous fat index in relation to overall survival at preoperative baseline, postoperative 2, 5 months.**

| Variable | Univariate Cox model | |  | Multivariate Cox model | |
| --- | --- | --- | --- | --- | --- |
|  | Crude HR (95% CI) | P value |  | Adjusted HR (95% CI) | P value |
| Preoperative baseline |  |  |  |  |  |
| SMI |  |  |  |  |  |
| Normal group | Ref |  |  | Ref |  |
| Low group | 1.31 (0.96, 1.80) | 0.088 |  | 1.31 (0.95, 1.81) | 0.096 |
| SFI |  |  |  |  |  |
| Normal group | Ref |  |  | Ref |  |
| High group | 1.28 (0.94, 1.74) | 0.121 |  | 1.26 (0.92,1.72) | 0.155 |
| Normal SMI and Normal SFI |  |  |  |  |  |
| Both | Ref |  |  | Ref |  |
| Neither | 1.42 (1.06, 1.90) | 0.017 |  | 1.39 (1.04, 1.87) | 0.027 |
| Postoperative 2 months |  |  |  |  |  |
| SMI |  |  |  |  |  |
| Normal group | Ref |  |  | Ref |  |
| Low group | 1.11 (0.77, 1.59) | 0.571 |  | 1.11 (0.77, 1.59) | 0.588 |
| SFI |  |  |  |  |  |
| Normal group | Ref |  |  | Ref |  |
| High group | 1.17 (0.81, 1.70) | 0.400 |  | 1.15 (0.79, 1.68) | 0.457 |
| Normal SMI and Normal SFI |  |  |  |  |  |
| Both | Ref |  |  | Ref |  |
| Neither | 1.21 (0.88, 1.66) | 0.231 |  | 1.18 (0.86, 1.63) | 0.300 |
| Postoperative 5 months |  |  |  |  |  |
| SMI |  |  |  |  |  |
| Normal group | Ref |  |  | Ref |  |
| Low group | 1.10 (0.70, 1.74) | 0.673 |  | 1.1 (0.69, 1.75) | 0.701 |
| SFI |  |  |  |  |  |
| Normal group | Ref |  |  | Ref |  |
| High group | 1.03 (0.67, 1.59) | 0.889 |  | 0.93 (0.59, 1.45) | 0.749 |
| Normal SMI and Normal SFI |  |  |  |  |  |
| Both | Ref |  |  | Ref |  |
| Neither | 1.07 (0.74, 1.56) | 0.704 |  | 1.00 (0.68, 1.46) | 0.980 |

Note: CI, confidence interval; HR, hazard ratio; Ref, reference; BMI, Body Mass Index; ECOG, eastern cooperative oncology group; SMI, skeletal muscle index; SFI, subcutaneous fat index;

Multivariate Cox model was adjusted for ECOG, pathological stage, tumor differentiation, lymph vascular invasion, perineural invasion, tumor deposit and adjuvant chemotherapy.
